# Prognostic implications of serum ferritin levels in non-anemic women with stage 3 chronic kidney disease

**DOI:** 10.3389/fnut.2025.1682003

**Published:** 2025-12-08

**Authors:** Min-Tser Liao, Chien-Lin Lu, Yi-Chou Hou, Joshua Wang, Kuo-Wang Tsai, Li-Jane Shih, Chia-Chao Wu, Yi-Chun Wang, Kuo-Cheng Lu

**Affiliations:** 1 Department of Pediatrics, Taoyuan Armed Forces General Hospital, Taoyuan, Taiwan; 2 Department of Pediatrics, Tri-Service General Hospital, National Defense Medical University, Taipei, Taiwan; 3 School of Medicine, College of Medicine, Fu Jen Catholic University, New Taipei City, Taiwan; 4 Division of Nephrology, Department of Medicine, Fu Jen Catholic University Hospital, New Taipei City, Taiwan; 5 Division of Nephrology, Department of Internal Medicine, Cardinal-Tien Hospital, School of Medicine, College of Medicine, Fu Jen Catholic University, New Taipei City, Taiwan; 6 Department of Research, Taipei Tzu Chi Hospital, Buddhist Tzu Chi Medical Foundation, New Taipei City, Taiwan; 7 Department of Medical Laboratory, Taoyuan Armed Forces General Hospital, Longtan, Taoyuan, Taiwan; 8 Graduate Institute of Medical Science, National Defense Medical Center, Taipei, Taiwan; 9 Division of Nephrology, Department of Internal Medicine, Tri-Service General Hospital, National Defense Medical Center, Taipei, Taiwan; 10 Division of Nephrology, Department of Medicine, Taipei Tzu Chi Hospital, Buddhist Tzu Chi Medical Foundation, New Taipei City, Taiwan; 11 Division of Nephrology, Department of Medicine, Buddhist Tzu Chi University, Hualien, Taiwan; 12 Division of Nephrology, Department of Medicine, Fu Jen Catholic University Hospital, School of Medicine, Fu Jen Catholic University, New Taipei City, Taiwan

**Keywords:** CKD, female, ferritin, non-anemic iron deficiency, AKI

## Abstract

**Introduction:**

Chronic kidney disease (CKD) is often accompanied by iron deficiency and persistent inflammation, both of which complicate the evaluation of iron metabolism and its clinical relevance. Although serum ferritin is commonly used to assess iron status in anemic CKD patients, data on its prognostic value in non-anemic, iron-deficient individuals remain limited and inconclusive.

**Methods:**

This retrospective cohort study utilized the TriNetX database to evaluate 5-year clinical outcomes in adult women with stage 3 chronic kidney disease (CKD), normal hemoglobin levels (≥12 g/dL), normal mean corpuscular volume (MCV, 80–100 fL), and varying serum ferritin concentrations. Patients were stratified into two groups based on ferritin levels: <100 ng/ml (low ferritin) and 100–700 ng/ml (adequate ferritin). Primary outcomes included all-cause mortality, major adverse cardiovascular events (MACE), acute kidney injury (AKI), pneumonia, fractures, and progression to advanced CKD (estimated glomerular filtration rate < 30 ml/min/1.73 m^2^).

**Results:**

A total of 66,768 eligible non-anemic women with stage 3 CKD, low serum ferritin levels, and normal MCV were identified. Propensity score matching (1,1) based on demographic variables was performed prior to comparing outcomes between low ferritin (*n* = 52,295) and adequate ferritin (*n* = 52,295) cohorts. Over 5 years, low ferritin—relative to adequate ferritin—was associated with significantly lower hazards of AKI, CKD progression, and pneumonia (HRs 0.909, 0.953, and 0.956; log-rank *p* < 0.05, <0.01, and <0.005, respectively). By contrast, low ferritin—relative to adequate ferritin—was associated with a significantly higher fracture hazard (HR 1.125; log-rank *p* < 0.05). No significant differences were observed in all-cause mortality or MACE. Low ferritin was associated with lower all-cause mortality at years 1–3 (ORs: 0.739, *p* < 0.001; 0.842, *p* < 0.05; 0.895, *p* = 0.038) and to a lower cumulative incidence of CKD progression at years 2–5 (ORs: 0.888 at year 2, 0.898 at year 3, 0.907 at year 4, 0.914 at year 5; all *p* < 0.05). Subgroup analysis revealed that low ferritin levels were especially protective against AKI and pneumonia in patients with elevated CRP (>10 mg/L), postmenopausal women, and those aged 18–64. The association of low ferritin levels with enhanced renal function preservation was more pronounced in postmenopausal women, individuals with vitamin D ≥ 30 ng/ml, and patients with diabetes. Conversely, adequate ferritin was associated witha lower fracture risk in older adults (>65 years) and those with vitamin D < 20 ng/ml.

**Conclusion:**

This study of non-anemic female patients with stage 3 CKD found that adequate ferritin levels correlated with a heightened risk of AKI, renal disease progression, and pneumonia. In contrast, low ferritin levels were associated with a higher fracture risk but a lower likelihood of renal function deterioration.

## Introduction

Iron deficiency is a widespread condition, affecting approximately 25% of the general population and 30–45% of chronic kidney disease (CKD) patients (1). Although bone marrow iron staining is the gold standard for assessing iron stores, its invasiveness limits routine use. Consequently, serum ferritin and transferrin saturation (TSAT) are widely used biomarkers—ferritin reflecting iron storage and TSAT indicating iron availability. Iron is vital for hemoglobin and myoglobin function, DNA synthesis, enzymatic activity, and mitochondrial energy production (2). Ferritin functions not only as an iron storage protein but also as an acute-phase reactant, rising in response to inflammation, malignancy, and systemic disease. Elevated ferritin has been associated with poor outcomes in sepsis (3), acute myocardial infarction (4), cerebrovascular disease (5), and rheumatoid disorders (6), highlighting its dual role as a marker of both iron status and inflammation. To better distinguish these effects, future studies should incorporate inflammatory markers such as CRP and IL-6, along with immunomodulators like vitamin D, to determine whether adverse outcomes are driven by iron imbalance or underlying inflammation (7).

The KDIGO guidelines recommend initiating iron therapy in non-dialysis CKD patients when ferritin is <100 ng/ml with transferrin saturation (TSAT) < 40%, or ferritin is 100–300 ng/ml with TSAT <25%. Iron supplementation is generally avoided if ferritin >700 ng/ml or TSAT >40% (8). A large retrospective study of 18,878 non-dialysis-dependent-CKD patients found no link between ferritin levels and mortality in females, while in males, ferritin <100 ng/ml was associated with a 23% increase in all-cause mortality, and TSAT ≤20% was associated with a 121% increase in all-cause mortality, suggesting that TSAT may be a superior prognostic marker (9). Another study found that low TSAT was a stronger predictor of mortality and MACE in NDD-CKD patients, while ferritin ≥300 ng/ml was associated withhigher mortality, with no consistent associations at lower ferritin levels (10). These findings underscore the limitations of ferritin as a standalone biomarker, as it may reflect inflammation rather than true iron status. Despite growing interest, data on iron deficiency in non-anemic CKD patients remain limited, and it is still unclear whether serum ferritin levels influence clinical outcomes in non-anemic female CKD patients.

We hypothesize that the protective effect of lower ferritin levels against adverse renal and infectious outcomes is not solely attributable to its role as an inflammatory marker but is rooted in fundamental iron biology (11). High iron stores can lead to excess labile iron, which catalyzes the Fenton reaction to generate reactive oxygen species, inducing oxidative stress that directly damages renal tubular cells and promotes fibrosis (12). Therefore, a state of lower iron storage, reflected by lower ferritin, may mitigate this iron-mediated nephrotoxicity and slow the progression of kidney disease (13). Furthermore, by restricting systemic iron availability, lower ferritin levels may enhance nutritional immunity, a host-defense strategy that limits pathogen access to essential iron, thereby reducing susceptibility to infections like pneumonia (14). This study aims to explore whether our observed clinical associations are consistent with these proposed biological mechanisms (15).

We assess the impact of serum ferritin levels below 100 ng/ml on five-year clinical outcomes in non-anemic women with stage 3 CKD, using ferritin levels between 100 and 700 ng/ml as the adequate control group. Using TriNetX data, it examines associations with all-cause mortality, major adverse cardiovascular events (MACE), acute kidney injury (AKI), pneumonia, fractures, and CKD progression (GFR ≤ 30 ml/min). These findings help to clarify the prognostic value of ferritin for clinical outcomes in patients with CKD.

## Patients and methods

### Study design

This retrospective cohort study was conducted by analysing data from the TriNetX network and adhered to Strengthening the Reporting of Observational Studies in Epidemiology (STROBE) guidelines. TriNetX is a global federated health research network^1^ providing access to electronic medical records—covering diagnoses, procedures, medications, laboratory results, and genomic data—from numerous large healthcare organizations (HCOs). This study used data from a subset of 139 HCOs within the Global Collaborative Network. The de-identification of patient data by TriNetX complies with Section §164.514(b)(1) of the HIPAA Privacy Rule (16). The study protocol was approved by the Institutional Review Board of Taipei Tzu Chi Hospital (Approval Number: 14-IRB027; approval date: 06/03/2025). 140 healthcare organizations were queried at the time of analysis using ICD-10 codes aligned with the study’s inclusion and exclusion criteria.

### Study cohorts

**Figure 1:**
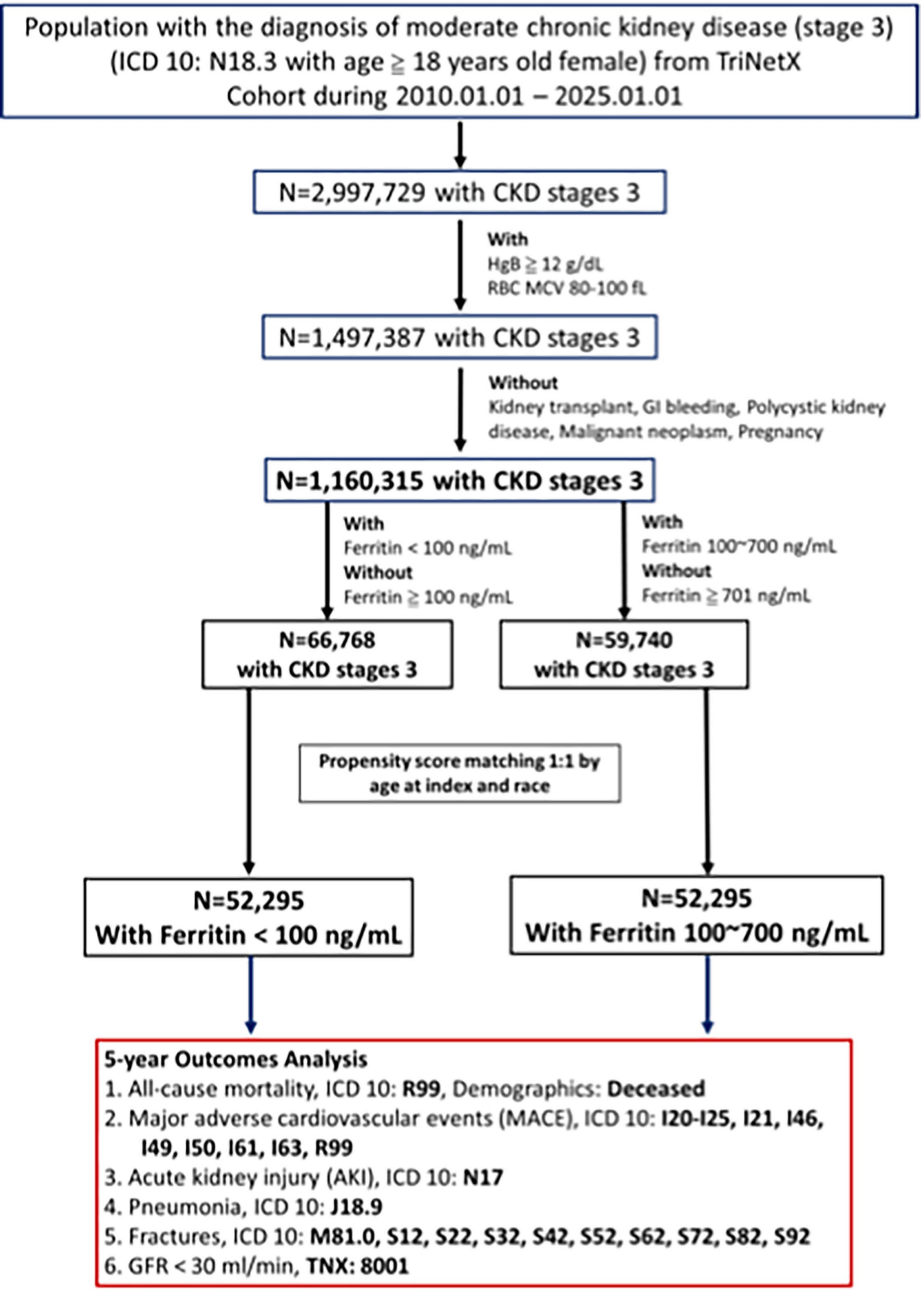
Flowchart outlining the patient selection process for the study.

Because ferritin reference intervals and diagnostic cut-offs for iron deficiency are sex specific (and under active re-evaluation), restricting to women avoids misclassification bias from applying mixed-sex thresholds (17, 18). Patient selection criteria, cohort characteristics, and the analytical methodology for assessing the long-term (5-year) outcomes in females with CKD stage 3 can be seen in Figure 1. This study included women aged ≥18 years with moderate CKD (ICD-10 N18.3), hemoglobin ≥12 g/dL, normal MCV (80–100 fL), and recorded serum ferritin levels between January 1, 2010, and January 1, 2025. Patients were excluded if they had a history of kidney transplantation (ICD-10 Z94.0), genitourinary malignancies (ICD-10 C51–C58, C64–C68), pregnancy (ICD-10 O00–O08, O09, O10–O16, O20–O29, O30–O48, O60–O77), or gastrointestinal bleeding (ICD-10 K92.2). Initially, 66,768 patients had low ferritin and 59,740 had adequate ferritin; after 1:1 propensity score matching, each group included 52,295 patients. Moderate CKD (stage 3) was defined using ICD-10 N18.3 and TriNetX code 8001, with eGFR calculated via the creatinine-based MDRD equation.

The study compares patients with a low ferritin group (<100 ng/ml) and an adequate ferritin group (100 ~ 700 ng/ml) follow the latest KDIGO guideline (8). To reduce the effect of demographic confounders, 1:1 propensity score matching was performed based on demographic factors. The primary outcomes assessed over 5 years include all-cause mortality (ICD-10: R99), major adverse cardiovascular events (MACE) (ICD-10: I20-I25, I21, I46, I49, I50, I61, I63, R99), acute kidney injury (ICD-10: N17), pneumonia (ICD-10: J18.9), fractures (ICD-10: M81.0, S12, S22, S32, S42, S52, S62, S72, S82, S92), and progression to GFR < 30 ml/min (TNX: 8001).

### Data analysis

The data analysis for this study involved evaluating clinical outcomes in propensity-matched cohorts of stage 3 CKD, non-anemic women with low or adequate serum ferritin levels over a follow-up period of 5 years. The presence of additional comorbidities or covariates such as diabetes mellitus, hypertension, menopause, age, 25-vitamin D and C-reactive protein (CRP) levels were also analyzed compared between the two cohorts.

To address the analytical limitations of the TriNetX platform, we implemented a two-stage analytical strategy. The first stage involved conducting an exploratory Cox proportional hazards analysis to screen for potential outcomes. Due to platform constraints, this preliminary model was adjusted for four principal confounders: age, sex, diabetes, and hypertension. Outcomes that demonstrated a statistically significant association in this exploratory model were advanced to a more comprehensive secondary analysis. In this stage, we performed Kaplan–Meier survival analysis and applied a Cox proportional hazards model that was fully adjusted for the complete set of covariates used for propensity score matching. This included detailed demographics (age, sex, race, ethnicity), as well as comorbidities, medications, and laboratory results. This two-step methodology allowed for an initial screening of relevant endpoints, which were then subjected to a more rigorously adjusted survival analysis.

In the Kaplan–Meier analysis, patients whose last recorded clinical event occurred within the observation period were censored by the TriNetX platform. The Kaplan–Meier survival data derived from the original TriNetX platform are presented in Supplementary Table 1. However, when calculating cohort risks for a defined timeframe, these patients are included rather than excluded. To account for this, we calculated the number of patients with the outcome and the corresponding risk for each 90-day interval (excluding repeated measures) and summed these across 21 intervals (90 × 21 = 1,890 days) to determine the total number of patients with the outcome over 5 years. Using these manually aggregated data, cumulative survival rates were computed using the Kaplan–Meier estimator to compare cohorts using the log-rank test. Raw data from the Kaplan–Meier analyses conducted on the TriNetX platform were extracted for each outcome, covering twenty-one 90-day intervals, and compiled into an Excel file (Supplementary Table 2). The survival analysis was then performed using the ‘survival’ package for R software (version 4.4.2, Vienna, Austria).

To assess statistical significance, independent two-sample t-tests were conducted, and the Bonferroni-Holm correction was applied to account for multiple comparisons across three tests, with adjusted *α* thresholds iteratively defined (e.g., α = 0.0083 for the smallest *p*-value of six tests). The six pre-specified primary outcomes were mortality, MACE, AKI, pneumonia, fractures, and CKD progression. Both unadjusted and Holm–Bonferroni adjusted *p*-values (e.g., α = 0.0083 as the smallest threshold across six tests) were calculated to control the family-wise error rate. Given the reduced sample size after propensity score matching, all reported statistically significant results had unadjusted p-values below their respective thresholds. Subsequent statistical analyses and data visualizations were performed using GraphPad Prism (Version 8.0) and SigmaPlot (Version 10.0).

#### Sensitivity analysis

To assess the robustness of our findings, we conducted two sensitivity analyses. First, we evaluated clinical outcomes across more granular ferritin ranges (100–300, 300–500, and 500–700 ng/ml). Second, to account for the influence of follow-up duration, we compared results from the primary cohort (enrolled 2010–2025) with those from a time-restricted cohort (enrolled 2010–2020), which ensured a potential follow-up period of at least 5 years for all patients.

#### Competing risk analysis and cumulative incidence curve

To account for the competing risk of mortality, we conducted a competing risk analysis on the TriNetX platform. This analysis provided the unadjusted cumulative incidence for pre-specified outcomes in each cohort, thereby isolating the event of interest from the influence of patient death. The platform utilizes the Aalen-Johansen estimator to generate cumulative incidence curves. By extracting the cumulative incidence values at defined follow-up intervals, we can constructed cumulative incidence curve.

## Results

### Patient characteristics

A total of 66,768 low ferritin patients and 59,740 adequate ferritin patients met the inclusion and exclusion criteria for the study. Table 1 provides a detailed comparison of patient baseline characteristics before and after propensity score matching. Demographic data, diagnosis comorbidity prevalence, medication usage, and laboratory values, were compared between patients with low or adequate ferritin levels. Before matching, the adequate ferritin group was older (63.4 ± 12.4 years vs. 59.6 ± 13.9 years, *p* < 0.001, standardized difference = 0.290) and had a lower proportion of White patients (70.9% vs. 62.6%, *p* < 0.001). Hypertensive diseases and Diabetes mellitus were significantly more prevalent in the adequate ferritin group (28.3% vs. 26.0%, *p* < 0.001; 10.9% vs. 10.3%, *p* < 0.001). To better balance biochemical test values, some laboratory variables were subdivided into multiple levels to help fully adjust for residual confounders. After matching, all demographic and laboratory differences were minimized, with standardized differences below 0.1, indicating that propensity score matching effectively eliminated demographic disparities between the two cohorts.

**Table 1:** Baseline patient characteristics before and after propensity score matching were reported.

Characteristics	Before Matching				After Matching			
	*F* < 100 (*n* = 66,768)	*F* ≥ 100 ~ 700 (*n* = 59,740)	*p* value	Std diff	*F* < 100 (*n* = 52,295)	*F* ≥ 100 ~ 700 (*n* = 52,295)	*p* value	Std diff
Demographics (%)								
Age at Index, mean ± SD	59.6 ± 13.9	63.4 ± 12.4	<0.001	0.290	62.6 ± 12.7	62.3 ± 12.4	<0.001	0.024
White (%)	70.9	62.6	<0.001	0.176	67.9	66.7	<0.001	0.025
Black or African American (%)	8.0	10.0	<0.001	0.069	8.8	9.3	0.005	0.017
Asian (%)	1.3	2.6	<0.001	0.095	1.6	1.7	0.072	0.011
Diagnosis (%)								
Diabetes mellitus	10.3	10.9	<0.001	0.021	10.8	11.2	0.041	0.013
Hypertensive diseases	26.0	28.3	<0.001	0.052	27.3	29.2	<0.001	0.043
Overweight and obesity	11.8	12.7	<0.001	0.026	11.0	13.7	<0.001	0.082
Ischemic heart diseases	4.4	4.8	0.008	0.015	4.8	4.8	1	<0.001
Cerebrovascular diseases	2.3	2.4	0.370	0.005	2.5	2.4	0.023	0.020
Medication (%)								
BETA BLOCKERS	11.5	12.4	<0.001	0.028	11.9	12.8	<0.001	0.027
ANTILIPEMIC AGENTS	16.6	16.7	0.506	0.004	17.9	17.1	0.003	0.019
CALCIUM CHANNEL BLOCKERS	8.2	8.7	0.003	0.017	8.9	8.6	0.111	0.010
ANGIOTENSIN II INHIBITOR	8.0	8.8	<0.001	0.027	8.7	8.9	0.299	0.006
ALPHA BLOCKERS	0.9	0.7	<0.001	0.020	0.6	0.5	0.031	0.019
Diuretics	13.9	15.4	<0.001	0.043	14.2	15.9	<0.001	0.047
Laboratory (mean ± SD)								
Sodium, mmol/L	139.5 ± 2.8	139.4 ± 3.2	<0.001	0.034	139.6 ± 2.9	139.4 ± 3.2	<0.001	0.079
Potassium, mmol/L	4.2 ± 0.7	4.2 ± 0.5	<0.001	0.041	4.2 ± 0.5	4.2 ± 0.5	<0.001	0.090
Calcium, mg/dL	9.4 ± 0.5	9.5 ± 0.6	<0.001	0.031	9.0 ± 0.8	8.8 ± 0.9	<0.001	0.052
Phosphate, mg/dL	3.6 ± 0.7	3.4 ± 0.7	<0.001	0174	3.6 ± 0.7	3.4 ± 0.7	0.002	0.130
0–3.50 mg/dL	3.3%	4.6%	<0.001	0.062	3.4%	4.5%	<0.001	0.057
3.50–5 mg/dL	3.4%	3.8%	<0.001	0.023	3.5%	3.8%	0.005	0.018
5–6.50 mg/dL	0.3%	0.3%	0.996	<0.001	0.3%	0.3%	0.856	0.001
>6.50 mg/dL	0.05%	0.1%	0.008	0.015	0.04%	0.1%	0.008	0.017
Magnesium, mg/dL	2.0 ± 0.3	2.0 ± 0.3	0.347	0.018	2.0 ± 0.3	2.0 ± 0.3	0.433	0.016
Hemoglobin, g/dL	13.8 ± 1.1	14.0 ± 1.2	<0.001	0.196	13.7 ± 1.1	14.0 ± 1.2	<0.001	0.228
10–11 g/dL	0.226%	0.122%	< 0.0001	0.0251	0.229%	0.12%	< 0.0001	0.0260
11–12 g/dL	0.548%	0.308%	< 0.0001	0.0367	0.54%	0.317%	< 0.0001	0.0341
12–13 g/dL	10.127%	9.769%	0.0401	0.0120	10.352%	9.639%	0.0002	0.0238
13–14 g/dL	17.206%	18.43%	< 0.0001	0.0320	17.189%	18.345%	< 0.0001	0.0303
14–15 g/dL	13.351%	16.401%	< 0.0001	0.0858	13.12%	16.538%	< 0.0001	0.0963
15–16 g/dL	5.506%	8.279%	< 0.0001	0.1096	5.382%	8.453%	< 0.0001	0.1213
16–17 g/dL	1.882%	3.047%	< 0.0001	0.0751	1.855%	3.153%	< 0.0001	0.0832
> 17 g/dL	0.705%	1.215%	< 0.0001	0.0523	0.676%	1.271%	0.0001	0.0605
Iron, ug/dL	79.8 ± 38.6	85.4 ± 38.3	<0.001	0.144	79.8 ± 36.9	85.5 ± 38.9	<0.001	0.152
0–50 ug/dL	1.4%	1.3%	0.127	0.009	1.3%	1.4%	0.545	0.004
50–100 ug/dL	4.0%	5.0%	<0.001	0.048	3.9%	5.1%	<0.001	0.057
100–150 ug/dL	1.5%	2.2%	<0.001	0.053	1.5%	2.3%	<0.001	0.060
>150 ug/dL	0.4%	0.5%	<0.001	0.027	0.3%	0.6%	<0.001	0.039
Ferritin, ng/ml	48.4 ± 33.9	180.1 ± 133.4	<0.001	1.352	49.2 ± 34.4	177.0 ± 133.2	<0.001	1.315
Creatinine, mg/dL	1.0 ± 0.5	1.0 ± 0.7	0.004	0.024	1.0 ± 0.4	1.0 ± 0.8	0.016	0.022
BUN, mg/dL	17.1 ± 6.6	18.1 ± 7.8	<0.001	0.131	17.6 ± 6.8	18.0 ± 7.7	<0.001	0.052
Bicarbonate, mmol/L	26.2 ± 3.3	25.9 ± 3.4	<0.001	0.098	26.3 ± 3.2	25.9 ± 3.4	<0.001	0.148
0–10 mmol/L	0.058%	0.078%	0.2234	0.0078	0.046%	0.073%	0.1160	0.0110
10–20 mmol/L	1.707%	2.119%	< 0.0001	0.0301	1.538%	2.193%	< 0.0001	0.0484
20–25 mmol/L	14.166%	14.961%	0.0004	0.0225	13.437%	16.291%	< 0.0001	0.0803
25–30 mmol/L	26.265%	26.457%	0.4563	0.0043	26.494%	27.331%	0.0029	0.0189
> 30 mmol/L	6.659%	6.201%	0.0014	0.0187	7.06%	6.225%	< 0.0001	0.0335
ALT, U/L	28.8 ± 44.3	40.2 ± 92.9	<0.001	0.156	28.3 ± 42.9	41.2 ± 96.8	<0.001	0.172
0–40 U/L	32.592%	33.556%	0.0004	0.0205	32.952%	33.49%	0.0715	0.0114
40–60 U/L	3.971%	6.055%	< 0.0001	0.0956	3.966%	6.253%	< 0.0001	0.104
60–80 U/L	1.684%	2.835%	< 0.0001	0.0775	1.638%	2.959%	< 0.0001	0.0882
80–100 U/L	0.802%	1.597%	< 0.0001	0.0731	0.757%	1.626%	< 0.0001	0.0802
100–120 U/L	0.482%	1.037%	< 0.0001	0.0640	0.458%	1.074%	< 0.0001	0.0707
120–140 U/L	0.307%	0.71%	< 0.0001	0.0567	0.293%	0.733%	< 0.0001	0.0616
140–160 U/L	0.2%	0.488%	< 0.0001	0.0492	0.195%	0.508%	< 0.0001	0.0529
>160 U/L	0.656%	1.404%	< 0.0001	0.0741	0.622%	1.461%	< 0.0001	0.0827
AST, U/L	28.1 ± 37.3	37.2 ± 75.5	<0.001	0.153	28.3 ± 38.7	37.6 ± 77.8	<0.001	0.153
0–40 U/L	32.028%	32.911%	0.0012	0.0189	32.055%	33.351%	< 0.0001	0.0276
40–60 U/L	3.555%	5.676%	< 0.0001	0.1012	3.579%	5.785%	< 0.0001	0.1046
60–80 U/L	1.22%	2.418%	< 0.0001	0.0897	1.245%	2.479%	< 0.0001	0.0914
80–100 U/L	0.556%	1.247%	< 0.0001	0.0732	0.564%	1.271%	< 0.0001	0.0742
100–120 U/L	0.308%	0.74%	< 0.0001	0.0598	0.309%	0.775%	< 0.0001	0.0635
120–140 U/L	0.19%	0.502%	< 0.0001	0.0531	0.197%	0.518%	< 0.0001	0.0538
140–160 U/L	0.13%	0.347%	< 0.0001	0.0446	0.126%	0.355%	< 0.0001	0.0467
>160 U/L	0.456%	1.085%	< 0.0001	0.0720	0.426%	1.116%	< 0.0001	0.0790
Alk phosphatase, U/L	84.6 ± 64.2	89.4 ± 48.7	<0.001	0.084	85.8 ± 69.9	89.3 ± 46.5	<0.001	0.059
Albumin, g/dL	4.1 ± 0.4	4.0 ± 0.5	<0.001	0.097	4.1 ± 0.4	4.0 ± 0.5	<0.001	0.056
Cholesterol, mg/dL	189.9 ± 45.0	190.2 ± 46.9	0.626	0.006	189.5 ± 44.8	190.5 ± 47.3	0.089	0.022
LDL Cholesterol, mg/dL	107.2 ± 38.1	108.7 ± 38.9	0.001	0.038	106.2 ± 38.2	109.0 ± 39.1	<0.001	0.072
Triglyceride, mg/dL	131.3 ± 76.8	140.8 ± 89.8	<0.001	0.113	130.0 ± 73.3	142.0 ± 90.2	<0.001	0.146
0–60 mg/dL	1.761%	1.43%	< 0.0001	0.0264	1.734%	1.455%	0.0004	0.0223
60–120 mg/dL	10.283%	10.97%	0.0001	0.0223	10.767%	10.839%	0.7133	0.0023
120–180 mg/dL	6.684%	7.423%	< 0.0001	0.0289	7.094%	7.523%	0.0092	0.0165
180–240 mg/dL	2.72%	3.351%	< 0.0001	0.0368	2.82%	3.394%	< 0.0001	0.0331
240–300 mg/dL	1.11%	1.333%	0.0005	0.0203	1.11%	1.403%	< 0.0001	0.0263
>300 mg/dL	0.91%	1.27%	< 0.0001	0.0346	0.893%	1.335%	< 0.0001	0.0421
Hemoglobin A1c, %	6.2 ± 1.3	6.3 ± 1.5	<0.001	0.059	6.3 ± 1.3	6.3 ± 1.5	0.037	0.029
Intact PTH, pg/ml	64.2 ± 53.2	63.1 ± 54.1	0.519	0.020	65.0 ± 55.1	63.2 ± 55.9	0.333	0.033
Calcidiol, ng/ml	39.2 ± 19.0	37.1 ± 18.4	<0.001	0.113	39.6 ± 19.1	37.4 ± 18.6	<0.001	0.122
0–20 ng/ml	0.8%	0.9%	0.008	0.015	0.8%	0.9%	0.039	0.013
20–30 ng/ml	1.4%	1.4%	0.997	<0.001	1.4%	1.4%	0.626	0.003
30–50 ng/ml	2.7%	2.4%	0.002	0.018	2.6%	2.4%	0.003	0.019
>50 ng/ml	1.5%	1.2%	<0.001	0.024	1.5%	1.2%	<0.001	0.026
CRP, mg/L	8.1 ± 18.5	19.5 ± 41.4	<0.001	0.355	8.2 ± 19.0	19.7 ± 41.3	<0.001	0.358
0–4 mg/L	4.0%	5.2%	<0.001	0.058	4.2%	4.9%	<0.001	0.038
4–10 mg/L	1.7%	2.7%	<0.001	0.071	1.7%	2.6%	<0.001	0.059
10–20 mg/L	0.9%	1.6%	<0.001	0.062	1.0%	1.5%	<0.001	0.052
20–30 mg/L	0.3%	0.8%	<0.001	0.066	0.3%	0.7%	<0.001	0.056
30–50 mg/L	0.3%	0.9%	<0.001	0.080	0.3%	0.8%	<0.001	0.068
50–80 mg/L	0.2%	0.7%	<0.001	0.082	0.2%	0.6%	<0.001	0.072
80–100 mg/L	0.1%	0.3%	<0.001	0.057	0.1%	0.3%	<0.001	0.051
100–130 mg/L	0.01%	0.3%	<0.001	0.063	0.01%	0.3%	<0.001	0.061
130–150 mg/L	0.01%	0.1%	<0.001	0.040	0.02%	0.1%	<0.001	0.036
>150 mg/L	0.01%	0.4%	<0.001	0.068	0.05%	0.3%	<0.001	0.062
Cobalamin	597.6 ± 436.1	626.7 ± 421.8	0.001	0.068	599.6 ± 368.5	628.0 ± 430.9	0.001	0.071
Folate	12.8 ± 8.1	12.1 ± 9.3	0.003	0.090	12.9 ± 8.3	12.2 ± 9.6	0.013	0.083

To improve balance in biochemical test values, some laboratory variables were categorized into multiple levels to better adjust for residual confounders. ALT, Alanine aminotransferase; AST, Aspartate aminotransferase; CRP, C reactive protein; PTH, Parathyroid hormone; Alk, Alkaline; BUN, Blood urea nitrogen; SD, standard deviation; Std. Diff., Standardized difference; MCV, mean corpuscular volume, F, Ferritin, ng/ml.

### The association of serum ferritin level with clinical outcomes

Cox proportional-hazards analysis (Supplementary Table 3).

Cox proportional-hazards models compared six outcomes between patients with ferritin <100 ng/ml and those with ferritin 100–700 ng/ml (reference group). Low ferritin was associated with lower risks of all-cause mortality (HR 0.897; 95% CI 0.832–0.968; *p* = 0.0048), acute kidney injury (HR 0.857; 95% CI 0.812–0.904; *p* < 0.0001), and pneumonia (HR 0.893; 95% CI 0.845–0.943; *p* < 0.0001). No significant difference was observed for progression to eGFR < 30 ml/min/1.73 m^2^ (HR 0.973; 95% CI 0.93–1.018; *p* = 0.2359). In contrast, low ferritin was associated withhigher risks of MACE (HR 1.042; 95% CI 1.018–1.067; *p* = 0.0007) and fractures (HR 1.236; 95% CI 1.202–1.27; *p* < 0.0001).

**Figure 2:**
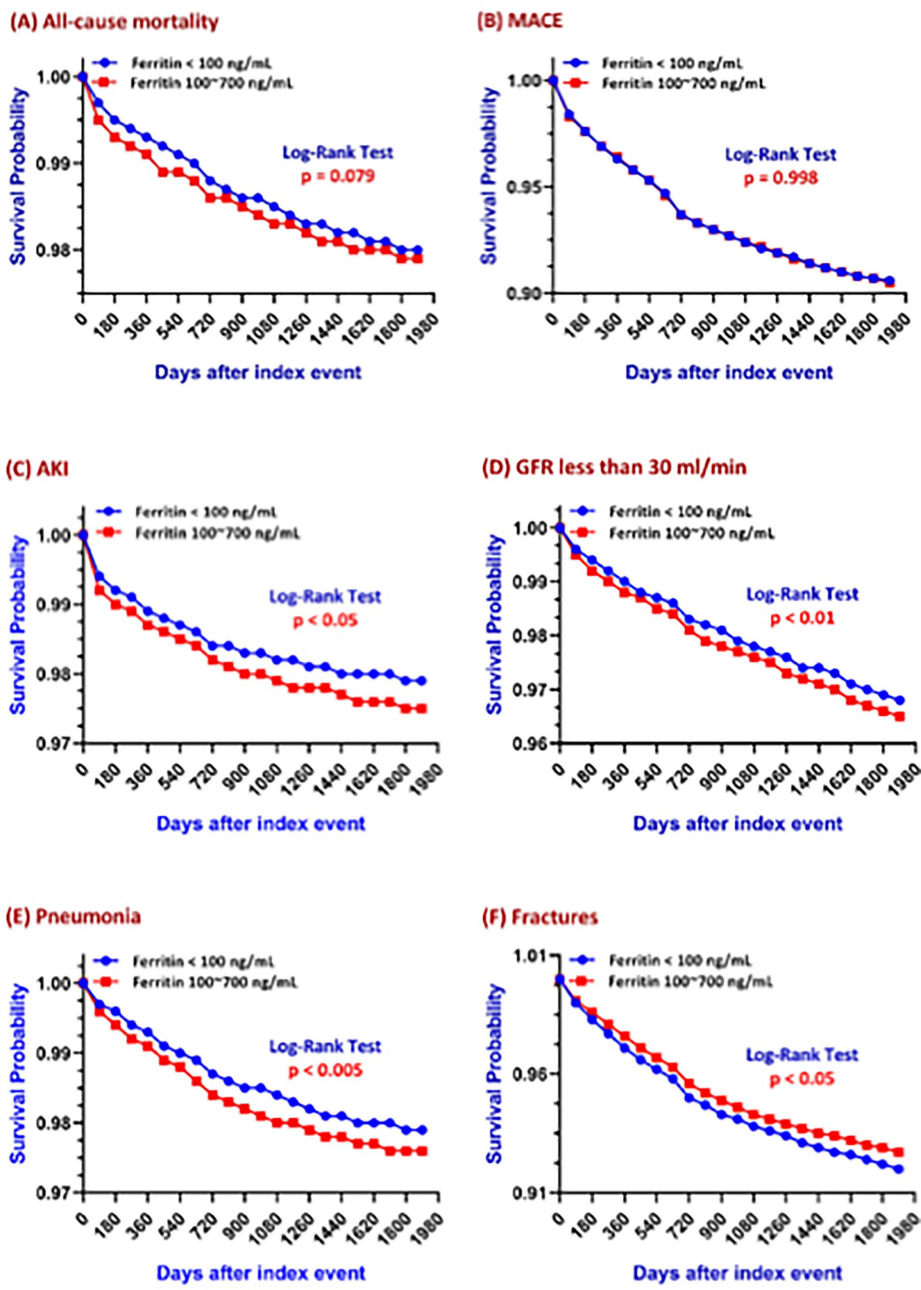
Five-year Kaplan–Meier survival curves showing the association between serum ferritin levels and clinical outcomes: **(A)** all-cause mortality, **(B)** major adverse cardiovascular events (MACE), **(C)** acute kidney injury (AKI), **(D)** GFR < 30 ml/min, **(E)** pneumonia, and **(F)** fractures, in patients stratified by ferritin levels (100–700 ng/ml vs. <100 ng/ml).

Kaplan–Meier survival analyses (Figure 2) demonstrated differential long-term risks between low ferritin and adequate ferritin patients. Over a 5-year follow-up:

#### All-cause mortality and MACE

**Figure 3:**
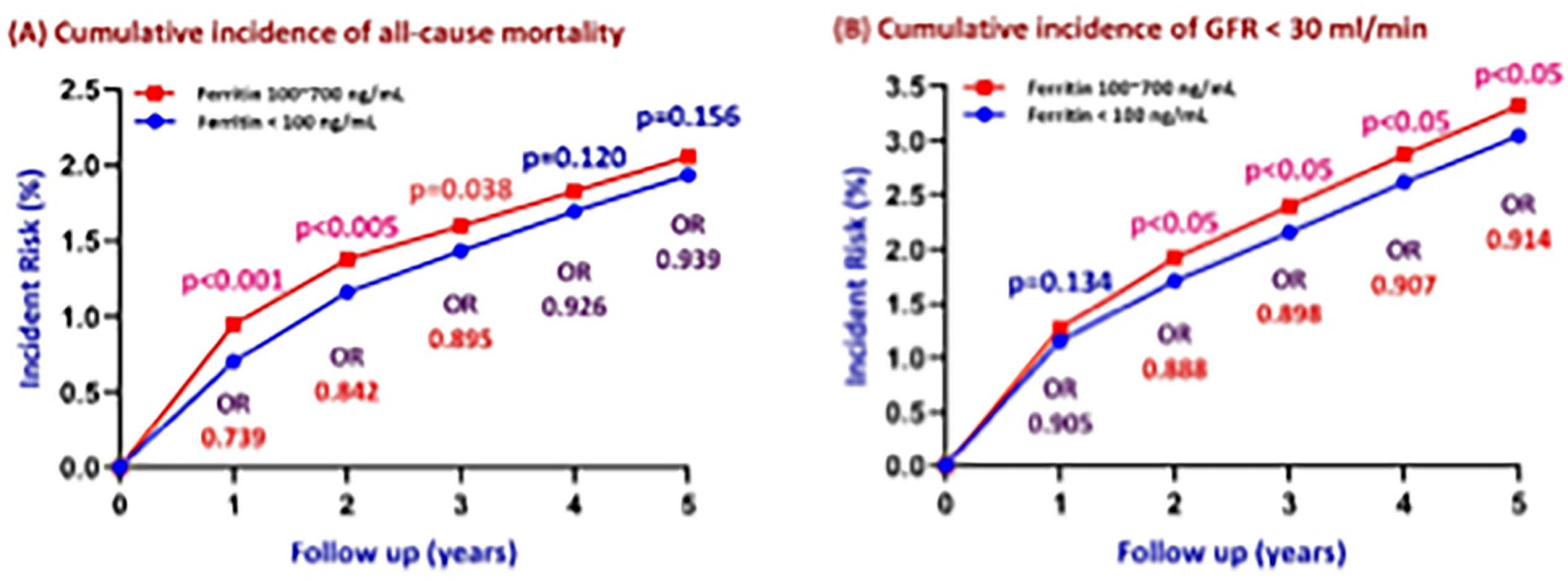
Association of serum ferritin levels with five-year cumulative incidence of **(A)** all-cause mortality and **(B)** renal function decline (GFR < 30 ml/min). *P*-values and odds ratios (ORs) reflect comparisons between ferritin levels of 100–700 ng/ml and <100 ng/ml at each annual follow-up.

Overall risk for all-cause mortality and MACE across the 5-year observation period were not statistically significantly different between the two groups (Figures 2A,B). However, Kaplan–Meier analysis for all-cause mortality (Figure 2A) showed a clear separation starting at year 1, with the adequate ferritin group exhibiting a significantly higher mortality risk during the first 2 years of follow-up. As illustrated in Figure 3A, the cumulative incidence of all-cause mortality was consistently higher in the adequate ferritin group at year 1 (*p* < 0.001) and year 2 (*p* < 0.05), but the difference became non-significant beyond year 2 (year 3, *p* = 0.038; year 4, *p* = 0.120; year 5, *p* = 0.156). The difference in mortality risk between the two groups dissipated over time, with yearly odds ratios progressively converging on 1.

#### AKI and renal function decline

In patients with ferritin <100 ng/ml, the 5-year AKI risk was 0.020 versus 0.025 in those with ferritin 100–700 ng/ml, corresponding to a risk difference of −0.005 and an odds ratio of 0.807; the *p*-value for the risk difference was <0.001. Likewise, patients with ferritin levels below 100 ng/ml had a 5-year risk of 0.029 for their GFR falling below 30 ml/min, compared to a risk of 0.032 in those with ferritin levels between 100 and 700 ng/ml. This corresponds to a statistically significant risk difference of −0.004 (*p* = 0.001) and an odds ratio of 0.887.

Patients with ferritin levels below 100 ng/ml had a 5-year risk of 0.029 for their Kaplan–Meier survival analysis showed that log-rank *p*-values for both AKI and renal function decline were <0.05 & < 0.01 respectively, indicating significantly lower incidence rates in patients with lower ferritin levels (Figures 2C,D). CKD progression was defined as a decline in GFR to <30 ml/min/1.73 m^2^. Patients in the adequate ferritin group experienced a significantly faster decline in renal function (log-rank *p* < 0.01). As shown in Figure 3B, the cumulative incidence of CKD progression over the 5-year follow-up was consistently higher in the adequate ferritin group compared to the low ferritin group. Although the between-group difference was not significant at year 1 (*p* = 0.134), it became statistically significant from year 2 onward (*p* < 0.05). The difference in CKD progression risk between the two cohorts dissipated over time, with ORs of 0.888 at year 2, 0.898 at year 3, 0.907 at year 4, and 0.914 at year 5.

#### Pneumonia

In the ferritin <100 ng/ml group, the 5-year pneumonia risk was 0.018 versus 0.022 in the 100–700 ng/ml group, yielding an absolute risk difference of −0.003 and an odds ratio of 0.846 (*p* < 0.001 for the risk difference). Kaplan–Meier analysis showed a significantly lower incidence of pneumonia in patients with low ferritin levels (log-rank *p* < 0.05; Figure 2E).

#### Fractures

A 5-year fracture risk of 0.071 was observed in patients with ferritin levels below 100 ng/ml, representing a statistically significant increase compared to the 0.066 risk found in patients with ferritin levels of 100–700 ng/ml (risk difference: 0.005, *p* = 0.007; odds ratio: 1.075). Kaplan–Meier analysis showed a significantly higher fracture risk in the low ferritin group (*p* < 0.05; Figure 2F). A comparison of the cohort’s overall risk at 5-years follow-up confirmed this association, suggesting that low ferritin may contribute to increased fracture risk, possibly due to impaired bone metabolism related to iron deficiency.

### Subgroup analyses of clinical outcomes by ferritin levels (100–700 ng/ml vs. <100 ng/ml)

**Figure 4:**
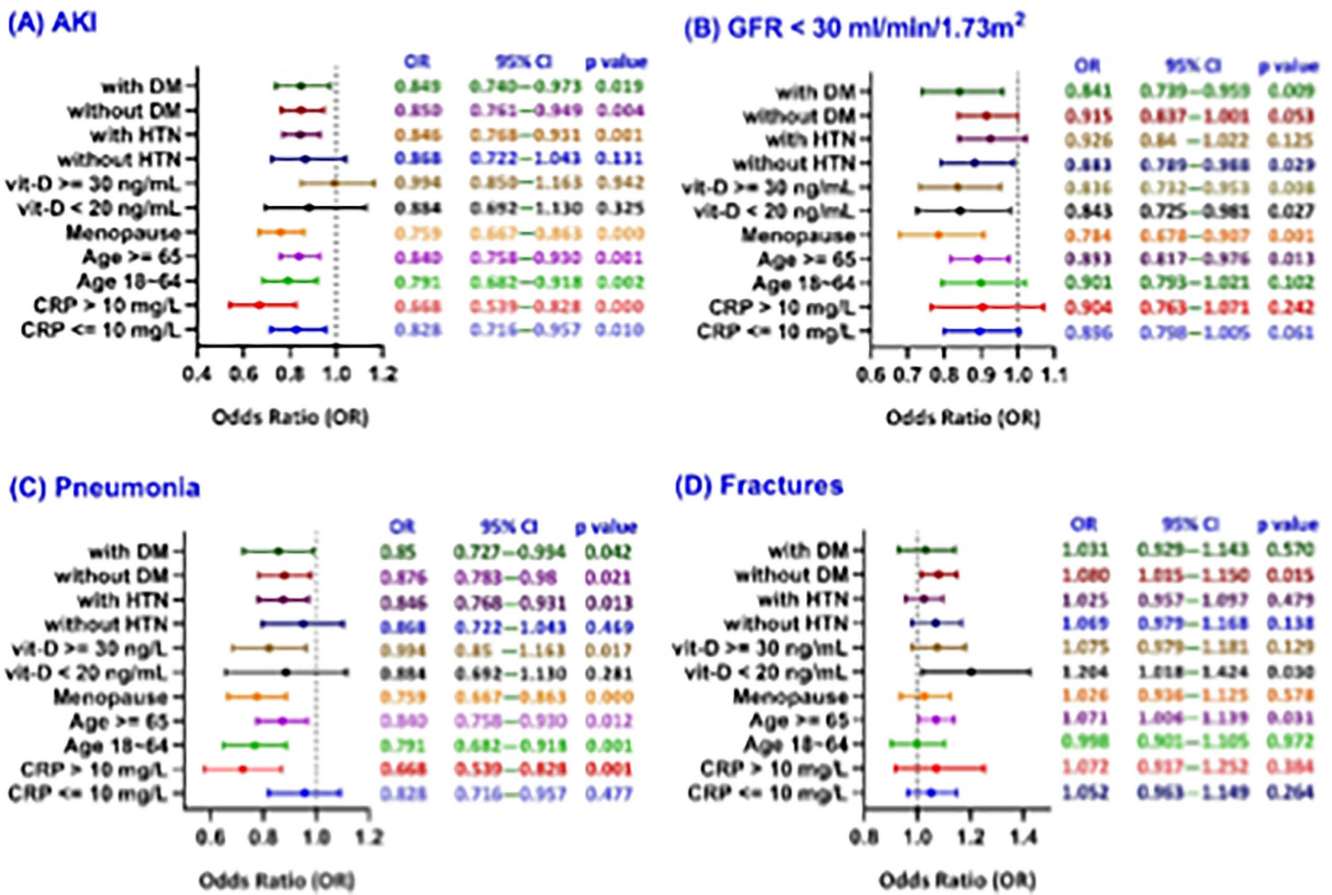
Forest plots illustrating subgroup analyses of selected covariates associated with specific clinical outcomes among patients stratified by serum ferritin levels (100–700 ng/ml vs. <100 ng/ml): **(A)** acute kidney injury (AKI), **(B)** GFR < 30 ml/min/1.73m², **(C)** pneumonia, and **(D)** fractures.

The forest plots present subgroup analyses of 5-year clinical outcomes—including acute kidney injury (AKI; Figure 4A), renal function decline (GFR < 30 ml/min; Figure 4B), pneumonia (Figure 4C), and fractures (Figure 4D) —in female patients with moderate CKD and normal hemoglobin levels. The analysis examines the influence of key variables such as CRP levels (≤10 vs. >10 mg/L), age (18–64 vs. ≥65 years), menopausal status, vitamin D levels (<20 vs. ≥30 ng/ml), and the presence or absence of hypertension (HTN) and diabetes (DM) on these outcomes.

#### Acute kidney injury and renal function decline

The analysis shows that lower ferritin levels were generally associated with reduced risks of AKI and renal function decline, suggesting a potential protective effect. The greatest reduction in AKI risk was observed in patients with elevated CRP (>10 mg/L), postmenopausal women, and individuals aged 18–64 years (Figure 4A). Similarly, the strongest protective effects against renal decline were seen in postmenopausal women, those with vitamin D ≥ 30 ng/ml, patients with diabetes, and older adults (Figure 4B).

#### Pneumonia

The low ferritin group was associated with a reduced risk of pneumonia across most subgroups (Figure 4C). The strongest protective effects were seen in patients with elevated CRP (>10 mg/L), younger age (18–64 years), and postmenopausal women. Notably, protection was also observed in those with vitamin D deficiency and diabetes, suggesting that systemic inflammation, hormonal status, and metabolic or nutritional factors may influence pneumonia risk in female CKD patients.

#### Fractures

The adequate ferritin group (100–700 ng/ml) was associated with a lower fracture risk, suggesting a protective role of iron stores in bone health (Figure 4D). The protective effect of adequate ferritin on fracture risk was significant in patients with vitamin D deficiency, older age (≥65), and without diabetes. No significant difference was observed in younger patients, indicating that age-related bone loss may be more influential than ferritin levels in fracture risk among younger CKD women.

Collectively, Kaplan–Meier analyses demonstrated that while the adequate-ferritin group had higher early mortality (first 2 years), this disparity attenuated over time (ORs ≈ 1). In contrast, associations with AKI, pneumonia, and renal decline remained significant throughout the 5-year follow-up. The effects were strongest in high-risk subgroups—elevated CRP (>10 mg/L), postmenopausal women, and younger patients—where low ferritin showed the clearest protective association. Although mortality differences weakened longitudinally, the consistent patterns across outcomes and subgroups support plausible inflammatory and iron-mediated mechanisms, warranting further prospective investigation.

### Sensitivity analysis of ferritin-level-dependent clinical outcome heterogeneity

To evaluate ferritin-dependent outcome variability, we performed sensitivity analyses using stratified ranges (100–300, 300–500, and 500–700 ng/ml), which yielded consistent results (Supplementary Table 4). In stage 3 CKD women without anemia, ferritin <100 ng/ml showed no mortality difference versus the 100–700 ng/ml reference. However, comparative analyses revealed significantly higher mortality hazards in the 301–500 and 501–700 ng/ml groups than in the <100 ng/ml cohort, indicating that hyperferitinemia may confer greater mortality risk than iron deficiency.

The second sensitivity analysis comparing the full (2010–2025) and time-restricted (2010–2020) enrollment cohorts was conducted to assess for heterogeneity in 5-year outcomes (Supplementary Table 5). The associations for mortality, AKI, eGFR decline, and pneumonia remained consistent, favoring the low-ferritin group. However, the MACE association shifted from neutral to modestly protective in the time-restricted analysis, while the elevated fracture risk seen in the full cohort was attenuated to null. Although absolute risks varied, the comparative hazard ratios for most outcomes remained stable, indicating minimal heterogeneity introduced by the enrollment period.

### Nutritional status and use of iron supplements

Nutritional status may be an unmeasured confounder affecting the association with low ferritin levels. To address this, we adjusted for baseline albumin levels post-propensity score matching, which partially mitigates, but does not fully eliminate, its influence. Regarding the potential protective effect of low ferritin, our hypothesis is supported by our recent publication (19). In that study, we found that iron supplementation in non-anemic women with stage 3 CKD and low ferritin was associated with increased risks of MACE, adverse renal events, and pneumonia, without offering a survival benefit. These findings reinforce the possibility that a low ferritin state may be protective in this population.

## Discussion

This study examined the relationship between serum ferritin levels and clinical outcomes in non-anemic, non-dialysis female patients with stage 3 CKD. Low ferritin levels (<100 ng/ml) were associated with reduced risks of AKI, renal decline, and pneumonia, particularly in postmenopausal women. Additionally, low ferritin was associated withlower all-cause mortality before year 2 and continued protection against renal decline beyond year 2. However, it was also associated with a higher risk of fractures, whereas adequate ferritin levels (100–700 ng/ml) were protective, especially in older adults and those with vitamin D deficiency. These findings underscore the complex role of ferritin in inflammation, bone health, and kidney function, demonstrating the importance of a personalized approach to CKD management.

Previous studies suggest that transferrin saturation (TSAT) is a more reliable predictor of mortality risk than serum ferritin. For instance, Guedes et al. found low TSAT, not ferritin, was more strongly associated with MACE, and serum ferritin ≥300 ng/ml showed no significant link to cardiovascular outcomes (3, 4). Rostoker et al. also highlighted inflammation as a key confounder affecting the interpretation of ferritin and TSAT, with significant impacts on mortality and cardiovascular outcomes in non-dialysis CKD patients (20). The study did not identify a statistically significant association between ferritin levels and 5-year all-cause mortality or major adverse cardiovascular events (MACE) (9, 10). Further analysis showed that low ferritin levels were associated with significantly lower mortality within the first 2 years, suggesting elevated ferritin—possibly reflecting inflammation—may contribute to short-term mortality in female CKD patients. These findings highlight the need for monitoring ferritin and investigating inflammatory markers to improve risk stratification and management.

In non-anemic CKD patients, the link between serum ferritin and kidney disease reflects the complex interaction of iron metabolism, inflammation, and kidney damage. A retrospective study in critically ill patients found ferritin levels >680 ng/ml were associated with higher 28-day mortality, regardless of sepsis, suggesting ferritin may indicate disease severity rather than cause injury directly (21). The Korean National Health Survey found high ferritin levels associated withincreased CKD risk in men but not women, suggesting a possible sex-specific susceptibility to renal decline (22). Consistent with prior studies, the findings indicated that adequate ferritin levels (100–700 ng/ml) were associated with a higher incidence of AKI, while low ferritin levels (<100 ng/ml) were associated witha reduced risk of AKI—particularly among postmenopausal women, individuals aged 18–64, and those with CRP > 10 mg/L—supporting the role of inflammation in increasing AKI risk.

Recent studies have clarified the link between ferritin and CKD progression. Tsai et al. found that elevated ferritin and hsCRP were independently associated with faster CKD progression and initiation of renal replacement therapy, with the highest ferritin tertile showing a 1.4-fold increased risk of renal decline (23). Eisenga et al. reported J- or U-shaped associations, with both low and high ferritin levels independently predicting CKD progression (24). Findings align with previous studies, indicating that female stage 3 CKD patients with adequate ferritin levels experienced greater renal decline over time, with the protective effect of low ferritin becoming most evident after year 2. This supports the role of inflammation in influencing ferritin’s impact, as CKD-related inflammation elevates hepcidin, promoting iron retention and ferritin synthesis. Pro-inflammatory cytokines like IL-1β and TNF-α further drive kidney damage by increasing ferritin expression and disrupting iron balance (25–27).

Ferroptosis, an iron-dependent form of regulated cell death driven by lipid peroxidation and mitochondrial dysfunction, has emerged as a key mechanism in AKI. Unlike apoptosis or necrosis, ferroptosis contributes to renal tubular injury in animal models through iron overload and oxidative stress (28). Clinically, this is relevant as nearly 50% of patients with hospital-associated AKI develop new-onset CKD within 3.3 years (29). The transition from AKI to CKD is primarily driven by maladaptive repair, involving proximal tubule injury, mitochondrial dysfunction, immune activation, and chronic inflammation (30). Emerging preclinical data suggest that inhibiting ferroptosis may protect mitochondrial function, reduce inflammation, and prevent tubular injury, offering potential benefits in diabetic nephropathy and AKI (31–34). These mechanisms may partly explain the long-term renal decline observed in this study. However, clinical evidence linking ferroptosis to AKI-to-CKD progression remains limited, warranting further investigation.

The link between serum ferritin and pneumonia risk in non-anemic CKD patients remains unclear due to limited direct evidence (22, 35). In CKD, elevated ferritin often reflects chronic inflammation, complicating the distinction between true iron overload and inflammation-driven hyperferritinemia (36). Emerging evidence suggests elevated ferritin may indicate inflammatory burden and increased infection risk, including infection by influenza and SARS-CoV-2 (37). In CKD, a pro-inflammatory state, high ferritin likely reflects immune dysregulation rather than iron overload (38, 39). Studies in critically ill patients further support this, linking hyperferritinemia to poorer sepsis outcomes, reinforcing its role as a marker of immune dysfunction (40). The study demonstrates a significant association between relatively high ferritin levels and increased pneumonia risk, while lower risk was observed in patients with reduced ferritin—especially among postmenopausal women, individuals with CRP > 10 mg/L, and those aged 18–64. Prior research suggests iron overload may raise infection risk by promoting bacterial growth, especially in CKD patients on iron therapy (41).

Lower ferritin levels (<100 ng/ml) were associated with an increased fracture risk in non-anemic CKD patients, particularly among those with low vitamin D, elevated CRP, and older age—suggesting a potential link between low ferritin and bone fragility. This aligns with prior research showing that iron deficiency impairs collagen synthesis and osteoblast function, reducing bone mineral density and increasing fracture risk (42). Iron deficiency leads to low bone turnover, impaired mineralization, and greater fracture risk, providing a mechanistic explanation for the increased fractures observed in the low-ferritin group (43). Patients with iron-deficiency anemia have also shown a higher risk of fractures, highlighting the essential role of iron in supporting skeletal integrity (44). Iron deficiency can impair bone metabolism, while excess iron may contribute to bone fragility through oxidative stress and reduced osteoblast activity (45). Additionally, CKD-related mineral and bone disorders independently affect bone health (46). These findings highlight the need for longitudinal studies incorporating bone turnover, inflammation, and mineral metabolism markers to clarify ferritin’s role in CKD-related bone fragility.

Our study reveals a critical trade-off associated with low ferritin levels. While a low ferritin state appears protective against adverse renal and infectious outcomes (47)—potentially through reduced oxidative stress and ferroptosis—it may simultaneously reflect an iron-deficient state that compromises bone health (43). This dichotomy underscores the clinical challenge of balancing the systemic benefits of lower iron stores against the potential risk to skeletal integrity.

In this retrospective cohort study, we collected baseline ferritin levels in a carefully selected population of stable, non-anemic women with stage 3 CKD, excluding participants with conditions known to affect ferritin metabolism (including kidney transplantation history, genitourinary malignancies, pregnancy, or active gastrointestinal bleeding). Following this cohort for 5 years ensured proper temporal sequencing between exposure and outcomes. To further address potential confounding, we performed 1:1 propensity score matching on demographic characteristics, clinical comorbidities, medication profiles, and relevant laboratory measures. This comprehensive approach significantly reduces concerns about reverse causation, a conclusion supported by the consistency of our findings across multiple sensitivity analyses.

### Limitations

This study has several important limitations. Its retrospective design using a large database network introduces inherent challenges, including the absence of transferrin saturation (TSAT) data, which precluded a full assessment of iron status, the potential for misclassification bias from diagnostic codes, and heterogeneity in laboratory assays across institutions. These issues are compounded by a high proportion of missing data for key laboratory values, particularly serum vitamin D (>60%) and C-reactive protein (≈90%), which reduced statistical power and introduced a significant risk of selection bias (Supplementary Table 6). Finally, the cohort was predominantly White and U.S.-based, which may limit the generalizability of our findings to other populations.

## Conclusion

This study underscores the multifaceted role of serum ferritin in non-anemic female patients with stage 3 CKD. Over 5 years, low ferritin levels (<100 ng/ml) were consistently associated withreduced risks of AKI, renal decline, and pneumonia—particularly in patients with inflammation, younger age, or postmenopausal status. Conversely, adequate ferritin levels (100–700 ng/ml) were associated with lower fracture risk, especially in older adults and those with vitamin D deficiency, suggesting a protective role in bone health. While overall mortality and MACE risks were comparable, early mortality was higher in the adequate ferritin group, possibly reflecting inflammatory burden. These findings highlight the need to interpret ferritin not only as an iron marker but also as an indicator of inflammation. Individualized ferritin targets based on age, comorbidities, inflammation, and bone health may improve clinical risk assessment. Further research is warranted to confirm these associations and inform iron management strategies.

## Data Availability

The original contributions presented in the study are included in the article/Supplementary material, further inquiries can be directed to the corresponding author.

## Footnotes

1
https://live.trinetx.com/

## References

[1] GBD Chronic Kidney Disease Collaboration. Global, regional, and national burden of chronic kidney disease, 1990-2017: a systematic analysis for the global burden of disease study 2017. Lancet (London, England). (2020) 395:709–33. doi: 10.1016/S0140-6736(20)30045-3, PMID: 32061315 PMC7049905

[2] Lopez A Cacoub P Macdougall IC Peyrin-Biroulet L. Iron deficiency anaemia. Lancet (London, England). (2016) 387:907–16. doi: 10.1016/S0140-6736(15)60865-0, PMID: 26314490

[3] Brandtner A Tymoszuk P Nairz M Lehner GF Fritsche G Vales A . Linkage of alterations in systemic iron homeostasis to patients' outcome in sepsis: a prospective study. J Intensive Care. (2020) 8:76. doi: 10.1186/s40560-020-00495-8, PMID: 33014378 PMC7528491

[4] Brinza C Floria M Popa IV Burlacu A. The prognostic performance of ferritin in patients with acute myocardial infarction: a systematic review. Diagnostics (Basel, Switzerland). (2022) 12:476. doi: 10.3390/diagnostics12020476, PMID: 35204567 PMC8870888

[5] Balla J Balla G Zarjou A. Ferritin in kidney and vascular related diseases: novel roles for an old player. Pharmaceuticals (Basel, Switzerland). (2019) 12:96. doi: 10.3390/ph12020096, PMID: 31234273 PMC6630272

[6] Seyhan S Pamuk Ö N Pamuk GE Çakır N. The correlation between ferritin level and acute phase parameters in rheumatoid arthritis and systemic lupus erythematosus. Eur J Rheumatol. (2014) 1:92–5. doi: 10.5152/eurjrheumatol.2014.032, PMID: 27708886 PMC5042227

[7] Fenercioglu AK Gonen MS Uzun H Sipahioglu NT Can G Tas E . The association between serum 25-hydroxyvitamin D3 levels and pro-inflammatory markers in new-onset type 2 diabetes mellitus and prediabetes. Biomolecules. (2023) 13:1778. doi: 10.3390/biom13121778, PMID: 38136648 PMC10741791

[8] Garabed Eknoyan NL Winkelmayer WC. KDIGO 2025 clinical practice guideline for anemia in chronic kidney disease (CKD). Kidney Int Suppl. (2025) 15:1–140. doi: 10.1016/j.kisu.2024.12.001

[9] Yu H Shao X Guo Z Pang M Chen S She C . Association of iron deficiency with kidney outcome and all-cause mortality in chronic kidney disease patients without anemia. Nutr J. (2025) 24:7. doi: 10.1186/s12937-025-01072-1, PMID: 39810180 PMC11734518

[10] Guedes M Muenz DG Zee J Bieber B Stengel B Massy ZA . Serum biomarkers of Iron stores are associated with increased risk of all-cause mortality and cardiovascular events in nondialysis CKD patients, with or without Anemia. J Am Soc Nephrol. (2021) 32:2020–30. doi: 10.1681/ASN.2020101531, PMID: 34244326 PMC8455257

[11] Dhondge RH Agrawal S Kumar S Acharya S Karwa V. A comprehensive review on serum ferritin as a prognostic marker in intensive care units: insights into ischemic heart disease. Cureus. (2024) 16:e57365. doi: 10.7759/cureus.57365, PMID: 38694418 PMC11061809

[12] Zhao Z. Iron and oxidizing species in oxidative stress and Alzheimer's disease. Aging Med (Milton). (2019) 2:82–7. doi: 10.1002/agm2.12074, PMID: 31942516 PMC6880687

[13] Xie T Yao L Li X. Advance in iron metabolism, oxidative stress and cellular dysfunction in experimental and human kidney diseases. Antioxidants (Basel). (2024) 13:659. doi: 10.3390/antiox13060659, PMID: 38929098 PMC11200795

[14] Shah AA Donovan K Seeley C Dickson EA Palmer AJR Doree C . Risk of infection associated with Administration of Intravenous Iron: a systematic review and Meta-analysis. JAMA Netw Open. (2021) 4:e2133935. doi: 10.1001/jamanetworkopen.2021.33935, PMID: 34767026 PMC8590171

[15] McCullough K Bolisetty S. Ferritins in kidney disease. Semin Nephrol. (2020) 40:160–72. doi: 10.1016/j.semnephrol.2020.01.007, PMID: 32303279 PMC7172005

[16] Palchuk MB London JW Perez-Rey D Drebert ZJ Winer-Jones JP Thompson CN . A global federated real-world data and analytics platform for research. JAMIA Open. (2023) 6:ooad035. doi: 10.1093/jamiaopen/ooad035, PMID: 37193038 PMC10182857

[17] Truong J Naveed K Beriault D Lightfoot D Fralick M Sholzberg M. The origin of ferritin reference intervals: a systematic review. Lancet Haematol. (2024) 11:e530–9. doi: 10.1016/S2352-3026(24)00103-0, PMID: 38937026

[18] Jager L Rachamin Y Senn O Burgstaller JM Rosemann T Markun S. Ferritin cutoffs and diagnosis of Iron deficiency in primary care. JAMA Netw Open. (2024) 7:e2425692. doi: 10.1001/jamanetworkopen.2024.25692, PMID: 39102268 PMC11301556

[19] Chen HC Liao MT Wang J Tsai KW Wu CC Lu KC. Clinical outcomes of Iron supplement therapy in non-Anemic female CKD stage 3 patients with low serum ferritin level: a multi-institutional TriNetX analysis. J Clin Med. (2025) 14:5575. doi: 10.3390/jcm14155575, PMID: 40807197 PMC12347412

[20] Rostoker G Lepeytre F Rottembourg J. Inflammation, serum iron, and risk of mortality and cardiovascular events in nondialysis CKD patients. J Am Soc Nephrol. (2022) 33:654–5. doi: 10.1681/ASN.2021081044, PMID: 35046130 PMC8975073

[21] Ren X Jiang Z Liu F Wang Q Chen H Yu L . Association of serum ferritin and all-cause mortality in AKI patients: a retrospective cohort study. Front Med. (2024) 11:1368719. doi: 10.3389/fmed.2024.1368719, PMID: 38938379 PMC11208335

[22] Kang HT Linton JA Kwon SK Park BJ Lee JH. Ferritin level is positively associated with chronic kidney disease in Korean men, based on the 2010-2012 Korean national health and nutrition examination survey. Int J Environ Res Public Health. (2016) 13:1058. doi: 10.3390/ijerph13111058, PMID: 27801876 PMC5129268

[23] Tsai YC Hung CC Kuo MC Tsai JC Yeh SM Hwang SJ . Association of hsCRP, white blood cell count and ferritin with renal outcome in chronic kidney disease patients. PLoS One. (2012) 7:e52775. doi: 10.1371/journal.pone.0052775, PMID: 23300770 PMC3534111

[24] Fujisawa H Nakayama M Haruyama N Fukui A Yoshitomi R Tsuruya K . Association between iron status markers and kidney outcome in patients with chronic kidney disease. Sci Rep. (2023) 13:18278. doi: 10.1038/s41598-023-45580-8, PMID: 37880328 PMC10600187

[25] Torti FM Torti SV. Regulation of ferritin genes and protein. Blood. (2002) 99:3505–16. doi: 10.1182/blood.V99.10.3505, PMID: 11986201

[26] Lemos DR McMurdo M Karaca G Wilflingseder J Leaf IA Gupta N . Interleukin-1β activates a MYC-dependent metabolic switch in kidney stromal cells necessary for progressive tubulointerstitial fibrosis. J Am Soc Nephrol. (2018) 29:1690–705. doi: 10.1681/ASN.2017121283, PMID: 29739813 PMC6054344

[27] Wen Y Lu X Ren J Privratsky JR Yang B Rudemiller NP . KLF4 in macrophages attenuates TNFα-mediated kidney injury and fibrosis. J Am Soc Nephrol. (2019) 30:1925–38. doi: 10.1681/ASN.2019020111, PMID: 31337692 PMC6779357

[28] Ni L Yuan C Wu X. Targeting ferroptosis in acute kidney injury. Cell Death Dis. (2022) 13:182. doi: 10.1038/s41419-022-04628-9, PMID: 35210424 PMC8873203

[29] Bucaloiu ID Kirchner HL Norfolk ER Hartle JE 2nd Perkins RM. Increased risk of death and de novo chronic kidney disease following reversible acute kidney injury. Kidney Int. (2012) 81:477–85. doi: 10.1038/ki.2011.405, PMID: 22157656

[30] Sato Y Takahashi M Yanagita M. Pathophysiology of AKI to CKD progression. Semin Nephrol. (2020) 40:206–15. doi: 10.1016/j.semnephrol.2020.01.011, PMID: 32303283

[31] Sui M Xu D Zhao W Lu H Chen R Duan Y . CIRBP promotes ferroptosis by interacting with ELAVL1 and activating ferritinophagy during renal ischaemia-reperfusion injury. J Cell Mol Med. (2021) 25:6203–16. doi: 10.1111/jcmm.16567, PMID: 34114349 PMC8256344

[32] Sha W Hu F Xi Y Chu Y Bu S. Mechanism of ferroptosis and its role in type 2 diabetes mellitus. J Diabetes Res. (2021) 2021:9999612. doi: 10.1155/2021/9999612 34258295 PMC8257355

[33] Wang WJ Jiang X Gao CC Chen ZW. Salusin-β participates in high glucose-induced HK-2 cell ferroptosis in a Nrf-2-dependent manner. Mol Med Rep. (2021) 24:12313. doi: 10.3892/mmr.2021.12313, PMID: 34296310 PMC8335735

[34] Wang J Liu Y Wang Y Sun L. The cross-link between ferroptosis and kidney diseases. Oxidative Med Cell Longev. (2021) 2021:6654887. doi: 10.1155/2021/6654887, PMID: 34007403 PMC8110383

[35] Kernan KF Carcillo JA. Hyperferritinemia and inflammation. Int Immunol. (2017) 29:401–9. doi: 10.1093/intimm/dxx031, PMID: 28541437 PMC5890889

[36] Ueda N Takasawa K. Impact of inflammation on ferritin, hepcidin and the Management of Iron Deficiency Anemia in chronic kidney disease. Nutrients. (2018) 10:1173. doi: 10.3390/nu10091173, PMID: 30150549 PMC6163440

[37] Hegelund MH Glenthøj A Ryrsø CK Ritz C Dungu AM Sejdic A . Biomarkers for iron metabolism among patients hospitalized with community-acquired pneumonia caused by infection with SARS-CoV-2, bacteria, and influenza. APMIS. (2022) 130:590–6. doi: 10.1111/apm.13259, PMID: 35751642 PMC9349447

[38] Ebert T Pawelzik SC Witasp A Arefin S Hobson S Kublickiene K . Inflammation and premature ageing in chronic kidney disease. Toxins. (2020) 12:227. doi: 10.3390/toxins12040227, PMID: 32260373 PMC7232447

[39] Rohr M Brandenburg V Brunner-La Rocca HP. How to diagnose iron deficiency in chronic disease: a review of current methods and potential marker for the outcome. Eur J Med Res. (2023) 28:15. doi: 10.1186/s40001-022-00922-6, PMID: 36617559 PMC9827648

[40] Schuster FS Nyvlt P Heeren P Spies C Adam MF Schenk T . Differential diagnosis of hyperferritinemia in critically ill patients. J Clin Med. (2022) 12:192. doi: 10.3390/jcm12010192, PMID: 36614993 PMC9821140

[41] Ganz T Aronoff GR Gaillard C Goodnough LT Macdougall IC Mayer G . Iron administration, infection, and anemia management in CKD: untangling the effects of intravenous iron therapy on immunity and infection risk. Kidney Med. (2020) 2:341–53. doi: 10.1016/j.xkme.2020.01.006, PMID: 32734254 PMC7380433

[42] Chon SJ Choi YR Roh YH Yun BH Cho S Choi YS . Association between levels of serum ferritin and bone mineral density in Korean premenopausal and postmenopausal women: KNHANES 2008-2010. PLoS One. (2014) 9:e114972. doi: 10.1371/journal.pone.0114972, PMID: 25522357 PMC4270774

[43] von Brackel FN Oheim R. Iron and bones: effects of iron overload, deficiency and anemia treatments on bone. JBMR Plus. (2024) 8:ziae064. doi: 10.1093/jbmrpl/ziae064, PMID: 38957399 PMC11215550

[44] Liu X An J. Dietary iron intake and its impact on osteopenia/osteoporosis. BMC Endocr Disord. (2023) 23:154. doi: 10.1186/s12902-023-01389-0, PMID: 37464304 PMC10353121

[45] Cai H Zhang H He W Zhang H. Iron accumulation and its impact on osteoporotic fractures in postmenopausal women. J Zhejiang Univ Sci B. (2023) 24:301–11. doi: 10.1631/jzus.B2200519, PMID: 37056206 PMC10106398

[46] Ketteler M Evenepoel P Holden RM Isakova T Jørgensen HS Komaba H . Chronic kidney disease-mineral and bone disorder: conclusions from a kidney disease: improving global outcomes (KDIGO) controversies conference. Kidney Int. (2025) 107:405–23. doi: 10.1016/j.kint.2024.11.013, PMID: 39864017

[47] Lyu G Liao H Li R. Ferroptosis and renal fibrosis: mechanistic insights and emerging therapeutic targets. Ren Fail. (2025) 47:2498629. doi: 10.1080/0886022X.2025.2498629, PMID: 40329437 PMC12057793

