# Supplementary material for: Prognostic implications of serum ferritin levels in non-anemic women with stage 3 chronic kidney disease
Source: Front Nutr. 2025 Dec 8;12:1682003. doi: 10.3389/fnut.2025.1682003 (PMC12723871; doi:10.3389/fnut.2025.1682003)
Supplement: Supplementary file 1 [file Table_1.docx]

**Supplemental Table 1.** Kaplan–Meier survival analysis data from TriNetX platform
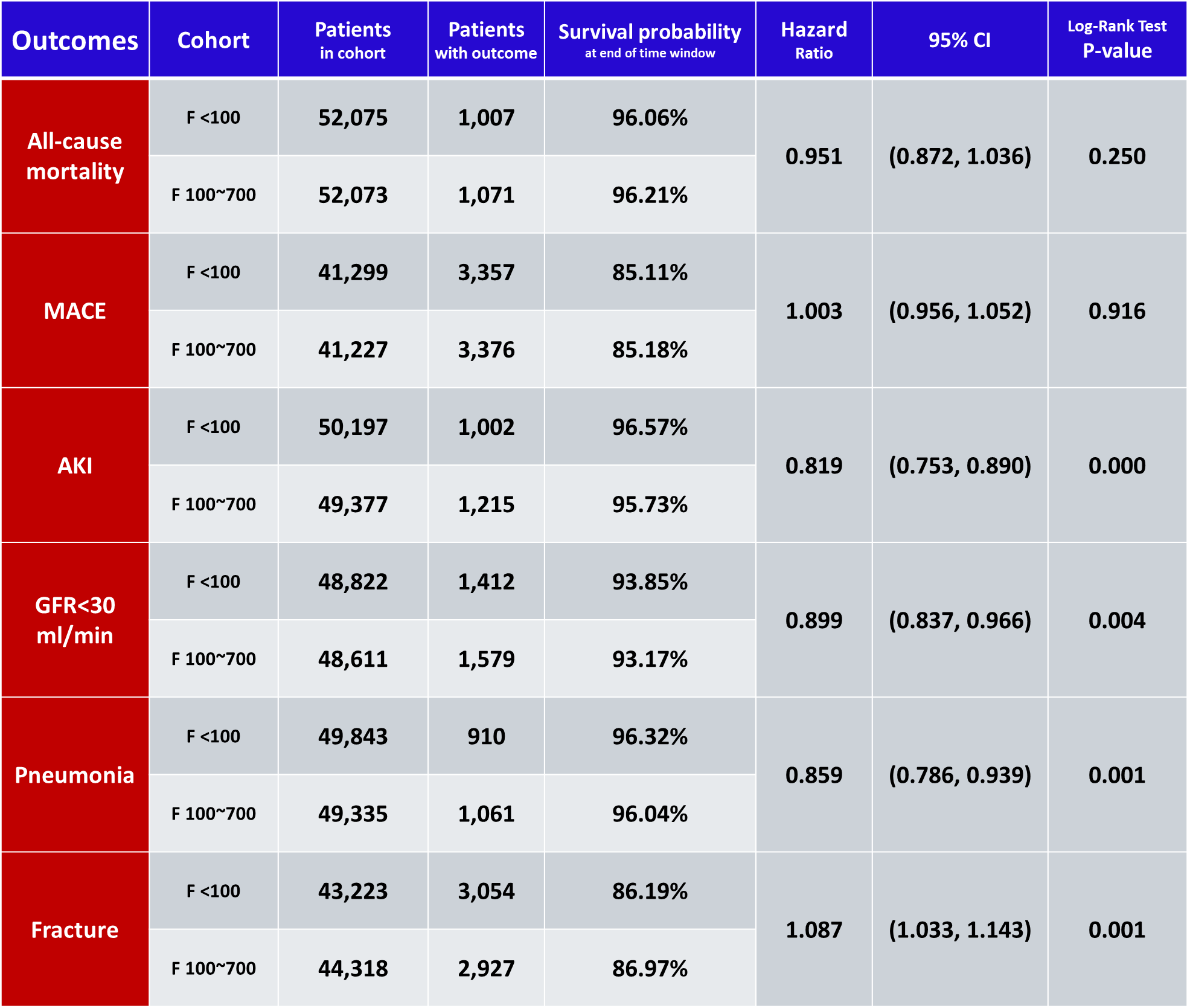


This survival analysis compared clinical outcomes for a cohort with ferritin levels of 100–700 ng/mL against a reference cohort with ferritin levels below 100 ng/mL. The study found no statistically significant difference in the risks for all-cause mortality (HR 0.951, p=0.250) or Major Adverse Cardiovascular Events (MACE) (HR 1.003, p=0.916). However, the F 100 cohort showed a significantly lower risk of developing Acute Kidney Injury (AKI) (HR 0.819, p=0.000), experiencing a GFR decline below 30 ml/min (HR 0.899, p=0.004), and developing pneumonia (HR 0.859, p=0.001). In contrast, this group faced a significantly higher risk of fractures (HR 1.087, p=0.001) when compared to the cohort with F 100~700 group.
