# Supplementary material for: Prognostic implications of serum ferritin levels in non-anemic women with stage 3 chronic kidney disease
Source: Front Nutr. 2025 Dec 8;12:1682003. doi: 10.3389/fnut.2025.1682003 (PMC12723871; doi:10.3389/fnut.2025.1682003)
Supplement: Supplementary file 2 [file Table_2.docx]

Raw data from the Kaplan-Meier analyses conducted on the TriNetX platform were extracted for each outcome, covering twenty-one 90-day intervals, and compiled into an Excel file (Supplemental Table 2). The survival analysis was then performed using the 'survival' package for R software (version 4.4.2, Vienna, Austria).

**Supplemental Table 2**
