# Supplementary material for: Prognostic implications of serum ferritin levels in non-anemic women with stage 3 chronic kidney disease
Source: Front Nutr. 2025 Dec 8;12:1682003. doi: 10.3389/fnut.2025.1682003 (PMC12723871; doi:10.3389/fnut.2025.1682003)
Supplement: Supplementary file 3 [file Table_3.docx]

**Supplemental Table 3.** Cox proportional-hazards models were used to estimate the hazard rates for various outcomes in patients with ferritin levels below 100 compared to those with ferritin levels between 100 ng/mL and 700 ng/mL

| **Cohort** | **Index count** | **Outcome** | **Hazard ratio** | **Coefficient** | **Standard Error** | **z** | **P > \|z\|** | **95% Confidence Interval** |
| --- | --- | --- | --- | --- | --- | --- | --- | --- |
| **F<100** | **74,881** | All-cause mortality | 0.897 | -0.108 | 0.038 | -2.817 | 0.0048 | (0.832, 0.968) |
| **F100~700** | **66,755** |  |  |  |  |  |  |  |
| **F<100** | **72,462** | MACE | 1.042 | 0.041 | 0.012 | 3.399 | 0.0007 | (1.018, 1.067) |
| **F100~700** | **66,003** |  |  |  |  |  |  |  |
| **F<100** | **68,577** | AKI | 0.857 | -0.154 | 0.027 | -5.663 | < 0.0001 | (0.812, 0.904) |
| **F100~700** | **64,555** |  |  |  |  |  |  |  |
| **F<100** | **68,256** | GFR<30 ml/min | 0.973 | -0.027 | 0.023 | -1.187 | 0.2353 | (0.93, 1.018) |
| **F100~700** | **64,305** |  |  |  |  |  |  |  |
| **F<100** | **75,783** | Pneumonia | 0.893 | -0.113 | 0.028 | -4.043 | < 0.0001 | (0.845, 0.943) |
| **F100~700** | **67,781** |  |  |  |  |  |  |  |
| **F<100** | **75,783** | Fracture | 1.236 | 0.212 | 0.014 | 15.132 | < 0.0001 | (1.202, 1.27) |
| **F100~700** | **67,781** |  |  |  |  |  |  |  |

**Cox proportional‑hazards results (Ferritin <100 ng/mL vs 100–700 ng/mL as reference)**

Compared with women whose ferritin was 100–700 ng/mL, those with ferritin <100 ng/mL had significantly lower hazards of all‑cause mortality (HR 0.90), AKI (HR 0.86), and pneumonia (HR 0.89), while showing no significant difference for progression to eGFR < 30 mL/min/1.73 m² (HR 0.97). Conversely, low ferritin was associated with higher hazards of MACE (HR 1.04) and fracture (HR 1.24).
