# Supplementary material for: Prognostic implications of serum ferritin levels in non-anemic women with stage 3 chronic kidney disease
Source: Front Nutr. 2025 Dec 8;12:1682003. doi: 10.3389/fnut.2025.1682003 (PMC12723871; doi:10.3389/fnut.2025.1682003)
Supplement: Supplementary file 4 [file Table_4.docx]

**Supplemental Table 4.**

***Sensitivity Analysis of Ferritin-Level-Dependent Clinical Outcome Heterogeneity***

1. **All-cause mortality**

| **Outcomes** | **Cohorts** | **Patients in cohort** | **Patients with outcome** | **Survival probability**  **at end of time window** | **Hazard Ratio** | **95% CI** | **Log-Rank test**  **P value** |
| --- | --- | --- | --- | --- | --- | --- | --- |
| **All-cause mortality** | **F <100** | **49,584** | **1,001** | **96.03%** | **0.946** | **(0.868, 1.031)** | **0.209** |
|  | **F: 100~700** | **49,605** | **1,061** | **96.17%** |  |  |  |
| **All-cause mortality** | **F <100** | **42,289** | **829** | **96.24%** | **1.053** | **(0.956, 1.161)** | **0.296** |
|  | **F: 100~300** | **42,324** | **789** | **96.56%** |  |  |  |
| **All-cause mortality** | **F <100** | **6,980** | **139** | **96.27%** | **0.554** | **(0.449, 0.682)** | **0.000** |
|  | **F: 301~500** | **6,979** | **243** | **93.46%** |  |  |  |
| **All-cause mortality** | **F <100** | **2,215** | **56** | **94.81%** | **0.462** | **(0.336, 0.636)** | **0.000** |
|  | **F: 501~700** | **2,217** | **116** | **91.82%** |  |  |  |

1. **MACE**

| **Outcomes** | **Cohorts** | **Patients in cohort** | **Patients with outcome** | **Survival probability**  **at end of time window** | **Hazard Ratio** | **95% CI** | **Log-Rank test**  **P value** |
| --- | --- | --- | --- | --- | --- | --- | --- |
| **MACE** | **F <100** | **38,546** | **3,485** | **83.97%** | **1.039** | **(0.991, 1.089)** | **0.116** |
|  | **F: 100~700** | **38,973** | **3,395** | **84.55%** |  |  |  |
| **MACE** | **F <100** | **33,211** | **2,971** | **84.35%** | **1.063** | **(1.009, 1.119)** | **0.021** |
|  | **F: 100~300** | **33,415** | **2,818** | **85.02%** |  |  |  |
| **MACE** | **F <100** | **5,543** | **460** | **85.31%** | **1.020** | **(0.895, 1.163)** | **0.763** |
|  | **F: 301~500** | **5,529** | **437** | **85.14%** |  |  |  |
| **MACE** | **F <100** | **1,743** | **163** | **82.54%** | **1.034** | **(0.826, 1.293)** | **0.771** |
|  | **F: 501~700** | **1,704** | **145** | **83.89%** |  |  |  |

1. **AKI**

| **Outcomes** | **Cohorts** | **Patients in cohort** | **Patients with outcome** | **Survival probability**  **at end of time window** | **Hazard Ratio** | **95% CI** | **Log-Rank test**  **P value** |
| --- | --- | --- | --- | --- | --- | --- | --- |
| **AKI** | **F <100** | **47,481** | **1,156** | **95.83%** | **0.925** | **(0.854, 1.002)** | **0.057** |
|  | **F: 100~700** | **46,988** | **1,239** | **95.35%** |  |  |  |
| **AKI** | **F <100** | **40,702** | **934** | **96.13%** | **0.972** | **(0.888, 1.063)** | **0.531** |
|  | **F: 100~300** | **40,487** | **958** | **95.93%** |  |  |  |
| **AKI** | **F <100** | **6,745** | **145** | **96.28%** | **0.711** | **(0.573, 0.882)** | **0.002** |
|  | **F: 301~500** | **6,565** | **192** | **94.98%** |  |  |  |
| **AKI** | **F <100** | **2,119** | **76** | **93.70%** | **1.016** | **(0.733, 1.410)** | **0.923** |
|  | **F: 501~700** | **1,991** | **68** | **94.43%** |  |  |  |

1. **GFR < 30 ml/min/1.73m^2^**

| **Outcomes** | **Cohorts** | **Patients in cohort** | **Patients with outcome** | **Survival probability**  **at end of time window** | **Hazard Ratio** | **95% CI** | **Log-Rank test**  **P value** |
| --- | --- | --- | --- | --- | --- | --- | --- |
| **GFR < 30 ml/min** | **F <100** | **46,076** | **1,495** | **93.28%** | **0.972** | **(0.905, 1.043)** | **0.431** |
|  | **F: 100~700** | **46,149** | **1,539** | **92.99%** |  |  |  |
| **GFR < 30 ml/min** | **F <100** | **39,238** | **1,333** | **93.12%** | **0.984** | **(0.912, 1.061)** | **0.676** |
|  | **F: 100~300** | **39,478** | **1,360** | **92.68%** |  |  |  |
| **GFR < 30 ml/min** | **F <100** | **6,438** | **234** | **92.50%** | **0.887** | **(0.743, 1.060)** | **0.188** |
|  | **F: 301~500** | **6,401** | **251** | **91.55%** |  |  |  |
| **GFR < 30 ml/min** | **F <100** | **2,051** | **82** | **91.12%** | **0.778** | **(0.579, 1.045)** | **0.094** |
|  | **F: 501~700** | **1,986** | **96** | **90.29%** |  |  |  |

1. **Pneumonia**

| **Outcomes** | **Cohorts** | **Patients in cohort** | **Patients with outcome** | **Survival probability**  **at end of time window** | **Hazard Ratio** | **95% CI** | **Log-Rank test**  **P value** |
| --- | --- | --- | --- | --- | --- | --- | --- |
| **Pneumonia** | **F <100** | **47,314** | **1,039** | **95.77%** | **0.952** | **(0.875, 1.037)** | **0.262** |
|  | **F: 100~700** | **46,995** | **1,086** | **95.80%** |  |  |  |
| **Pneumonia** | **F <100** | **40,313** | **818** | **96.12%** | **0.968** | **(0.880, 1.066)** | **0.512** |
|  | **F: 100~300** | **40,181** | **844** | **96.18%** |  |  |  |
| **Pneumonia** | **F <100** | **6,723** | **130** | **96.37%** | **0.804** | **(0.636, 1.016)** | **0.067** |
|  | **F: 301~500** | **6,576** | **153** | **95.25%** |  |  |  |
| **Pneumonia** | **F <100** | **2,118** | **57** | **94.88%** | **0.906** | **(0.628, 1.309)** | **0.600** |
|  | **F: 501~700** | **2,025** | **57** | **94.95%** |  |  |  |

1. **Fracture**

| **Outcomes** | **Cohorts** | **Patients in cohort** | **Patients with outcome** | **Survival probability**  **at end of time window** | **Hazard Ratio** | **95% CI** | **Log-Rank test**  **P value** |
| --- | --- | --- | --- | --- | --- | --- | --- |
| **Fractures** | **F <100** | **40,814** | **3,138** | **85.34%** | **1.117** | **(1.062, 1.175)** | **0.000** |
|  | **F: 100~700** | **42,153** | **2,925** | **86.63%** |  |  |  |
| **Fractures** | **F <100** | **35,076** | **2,613** | **85.94%** | **1.074** | **(1.017, 1.134)** | **0.011** |
|  | **F: 100~300** | **35,996** | **2,517** | **86.69%** |  |  |  |
| **Fractures** | **F <100** | **5,822** | **398** | **86.46%** | **1.232** | **(1.065, 1.426)** | **0.005** |
|  | **F: 301~500** | **6,081** | **329** | **89.24%** |  |  |  |
| **Fractures** | **F <100** | **1,811** | **122** | **87.49%** | **1.226** | **(0.939, 1.600)** | **0.133** |
|  | **F: 501~700** | **1,911** | **98** | **89.02%** |  |  |  |

In non-anemic women with stage 3 CKD, ferritin levels below 100 ng/mL showed no significant link to 5-year mortality compared to the 100–700 ng/mL range but were associated with lower mortality risk than higher ferritin subgroups (301–700 ng/mL), suggesting elevated ferritin may be more detrimental than iron deficiency. While low ferritin minimally affected cardiovascular risk, it significantly increased fracture risk, highlighting the need for bone health monitoring. Interestingly, low ferritin was tied to reduced acute kidney injury risk, whereas ferritin levels had no clear impact on CKD progression or pneumonia. These findings imply that high ferritin may pose greater mortality risks, whereas low ferritin primarily affects bone health, warranting tailored clinical attention.
