# Supplementary material for: Prognostic implications of serum ferritin levels in non-anemic women with stage 3 chronic kidney disease
Source: Front Nutr. 2025 Dec 8;12:1682003. doi: 10.3389/fnut.2025.1682003 (PMC12723871; doi:10.3389/fnut.2025.1682003)
Supplement: Supplementary file 5 [file Table_5.docx]

**Supplemental Table 5.** Sensitivity analysis by enrollment period was conducted to assess heterogeneity in 5-year clinical outcomes.


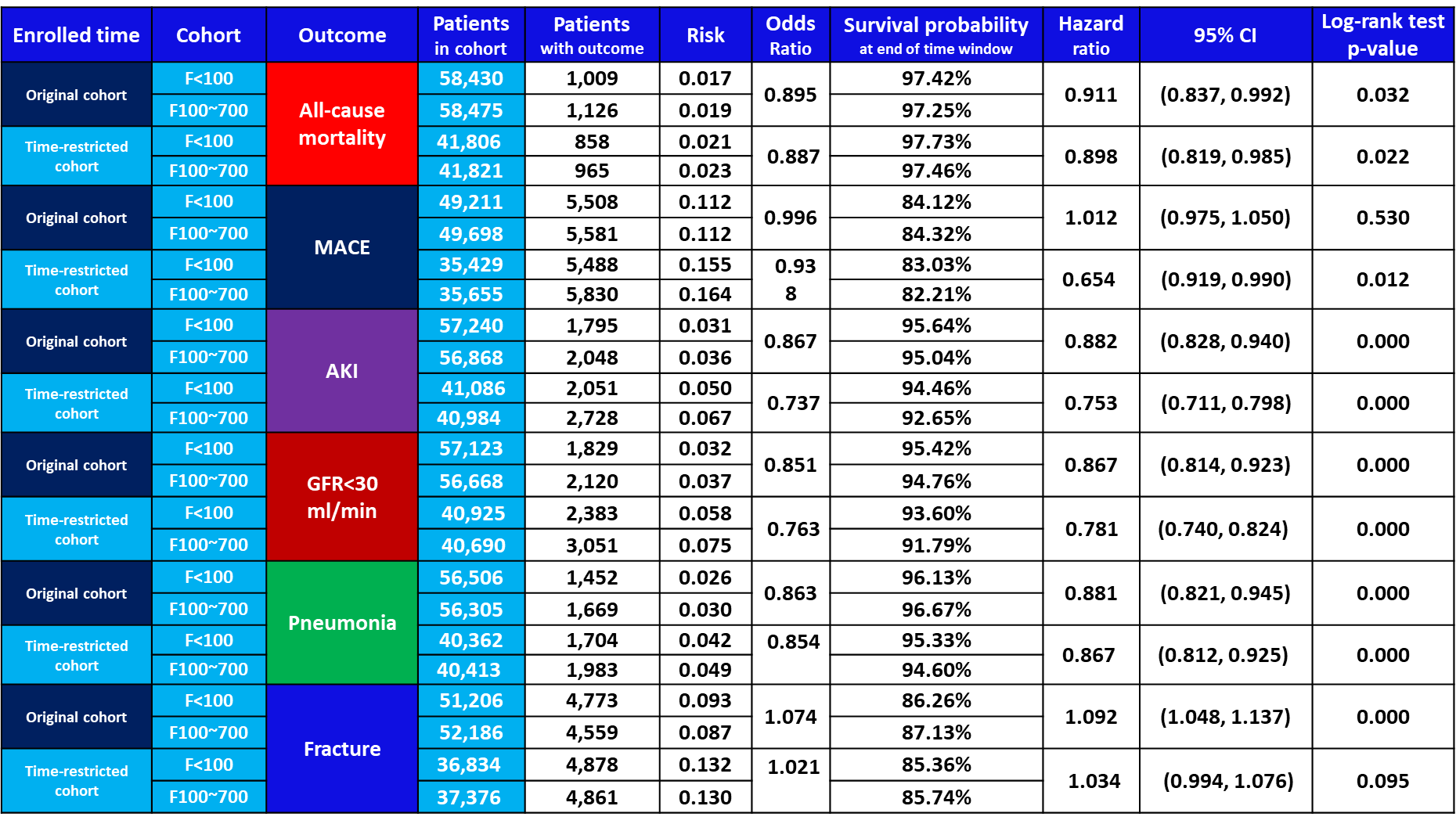


We compared the original cohort (enrolled January 1, 2010–January 1, 2025) with a time-restricted cohort (January 1, 2010–January 1, 2020) to ensure all patients in the latter had at least 5 years of follow-up. Risk analyses and Kaplan–Meier survival analyses were used to estimate hazard rates for predefined outcomes across these enrollment-period cohorts. For each endpoint we reported cohort size, events, risk, 5-year Kaplan–Meier survival, odds ratio, hazard ratio (HR) with 95% CI, and log-rank p-value. Results were directionally concordant across windows: all-cause mortality favored F<100 in both analyses (original HR 0.911, 95% CI 0.837–0.992; time-restricted HR 0.898, 0.819–0.985); MACE was null in the full window but showed a modest benefit with F<100 in the restricted analysis (HR ≈0.954, 0.919–0.990); AKI and progression to eGFR <30 mL/min/1.73 m² consistently favored F<100, with stronger effects in the restricted cohort (AKI HR 0.753, 0.711–0.798; eGFR<30 HR 0.781, 0.740–0.824); and pneumonia risk was lower with F<100 in both windows (HR 0.881, 0.821–0.945; and 0.867, 0.812–0.925). The fracture signal—elevated in the full window (HR 1.092, 1.048–1.137)—attenuated to non-significance with uniform follow-up (HR 1.034, 0.994–1.076), suggesting sensitivity to enrollment period or residual confounding. Overall, while absolute risks and 5-year survival differed as expected with longer uniform follow-up, comparative HRs were stable, indicating minimal heterogeneity by enrollment period for most outcomes.

Across enrollment windows, the direction and magnitude of effects were highly consistent for mortality, AKI, eGFR < 30 mL/min/1.73 m², and pneumonia, all favoring the F<100 group. MACE was neutral in the full-window analysis but demonstrated a modest protective association in the time-restricted cohort. By contrast, the apparent fracture excess observed in the full window attenuated to null with uniform follow-up, suggesting sensitivity to enrollment period or residual confounding. Although absolute risks and 5-year survival values shifted as expected in the time-restricted cohort, the comparative hazard ratios remained stable, indicating minimal heterogeneity by enrollment period for most outcomes.
