# Supplementary material for: Prognostic implications of serum ferritin levels in non-anemic women with stage 3 chronic kidney disease
Source: Front Nutr. 2025 Dec 8;12:1682003. doi: 10.3389/fnut.2025.1682003 (PMC12723871; doi:10.3389/fnut.2025.1682003)
Supplement: Supplementary file 6 [file Table_6.docx]

**Supplemental Table 6.** **Missing data in vit-D & CRP**. Subgroup analyses of AKI, eGFR <30 mL/min/1.73 m², pneumonia, and fracture risk across strata defined by vitamin D and CRP levels—25(OH)D ≥30 ng/mL, 25(OH)D <20 ng/mL, CRP >10 mg/L, and CRP ≤10 mg/L. In all subgroups, risks were compared between the F<100 and F100–700 cohorts.
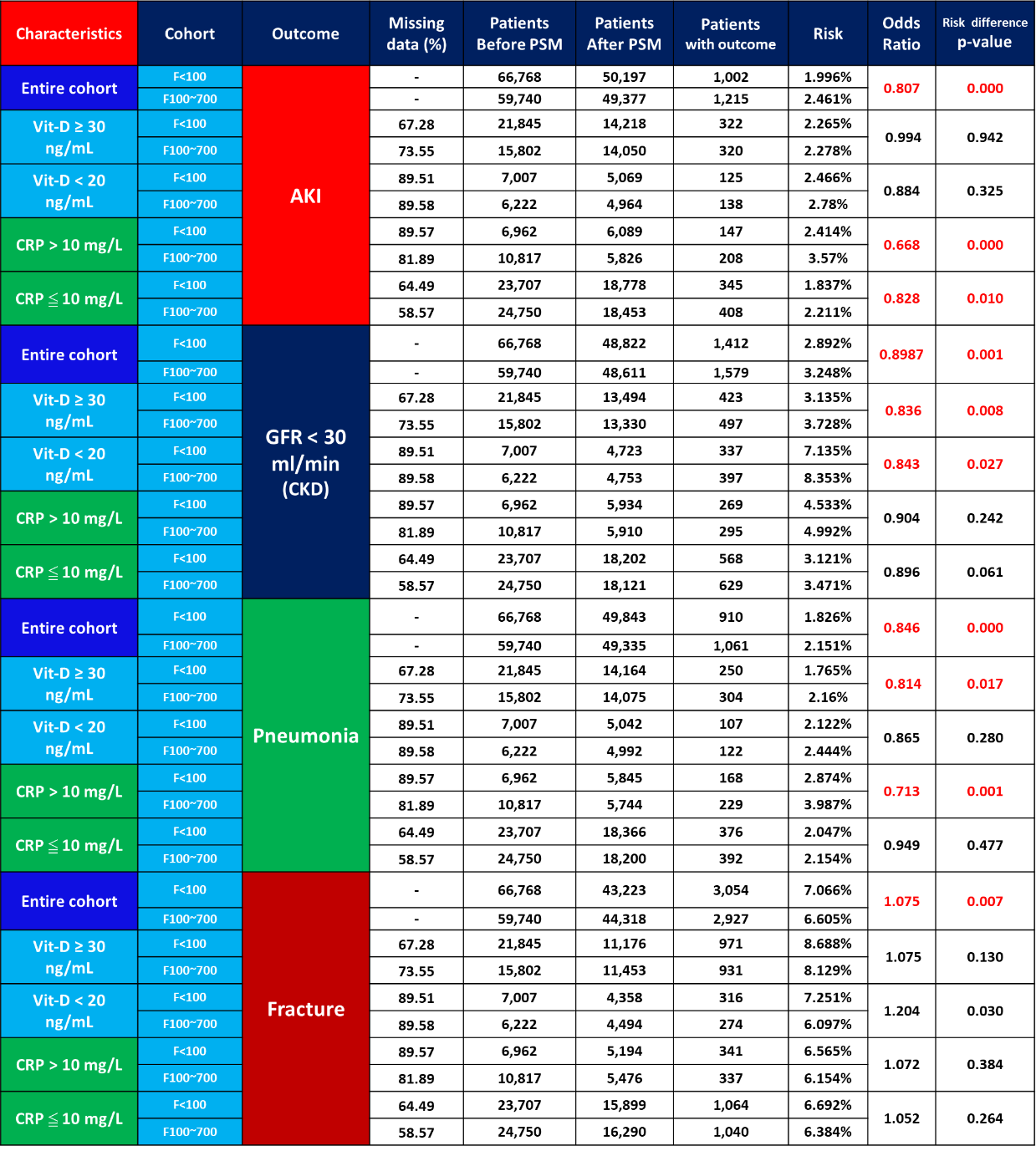


Across all four outcomes (AKI, eGFR < 30 mL/min/1.73 m², pneumonia, fracture), the entire cohort provides the largest analyzable samples (e.g., AKI before/after PSM: F<100 = 66,768→50,197; F100–700 = 59,740→49,377). In contrast, the vitamin-D and CRP strata are much smaller because many patients lack these labs. The table reports the same percent missing for each outcome: Vit-D ≥ 30 ng/mL: 67.28% missing (only 32.72% observed); Vit-D < 20 ng/mL: 73.55% missing (26.45% observed); CRP > 10 mg/L: 89.58% missing (10.42% observed); CRP ≤ 10 mg/L: 58.57% missing (41.43% observed). This attrition translates into markedly reduced subgroup sample sizes before PSM and then a further drop after PSM (illustratively for AKI, after PSM the subgroups are roughly: Vit-D ≥ 30 ng/mL ≈14–15k per arm, Vit-D < 20 ng/mL ≈4.9–5.0k vs ≈7.2–7.7k, CRP > 10 mg/L ≈5.0–6.0k per arm, CRP ≤ 10 mg/L ≈18–19k per arm—similar magnitudes across outcomes). Despite these reductions, the **direction of effect** (F<100 vs F100–700) generally mirrors the full-cohort results: lower odds for AKI and eGFR decline, and lower pneumonia risk in several strata; the fracture excess seen in the full cohort attenuates within strata.

**Clinical significance of the missing-data pattern**

High levels of missing data (≥60% for vitamin D and nearly 90% for CRP) mean subgroup analyses draw on a minority of the target population, reducing event counts, widening confidence intervals, and increasing the likelihood of non-significant results even when effects exist (e.g., fracture). Because patients with available Vit-D/CRP measurements may differ clinically (severity, monitoring intensity, comorbidities), estimates derived from these subgroups generalize primarily to “patients with labs,” introducing potential selection bias. Although PSM preserves covariate balance within the retained samples, the substantial attrition from pre- to post-PSM in smaller strata raises variance, limits overlap, and further constrains external validity. Accordingly, while the protective signals for AKI, eGFR decline, and—across several strata—pneumonia support an association favoring F<100, effect sizes should be interpreted cautiously; where signals appear weaker (e.g., fracture), diminished statistical power and selection effects are plausible contributors.

Although we report missingness rates, the TriNetX network platform limits our ability to compare characteristics of included versus excluded patients and to augment complete-case analyses with multiple imputation or inverse-probability weighting for lab availability. Consequently, complementary real-world clinical data collection is needed to address these limitations.
